# Green Tea Catechins Mitigate Hepatocyte Ferroptosis Through Attenuation of Oxidative Stress and Improvement of Antioxidant Systems

**DOI:** 10.3390/antiox14121483

**Published:** 2025-12-10

**Authors:** Pimpisid Koonyosying, Wit Tharanon, Kavee Pairojthanachai, Yanisa Samakarn, Kornkan Meejak, Narisara Paradee, Onsaya Kerdto, Suphatta Yubo, Yanping Zhong, Somdet Srichairatanakool

**Affiliations:** 1 Department of Biochemistry, Faculty of Medicine, Chiang Mai University, Chiang Mai 50200, Thailand; pimpisid.k@cmu.ac.th (P.K.); wthara@tu.ac.th (W.T.); kavee.pairojtanachai@cmu.ac.th (K.P.); yanisa.s@cmu.ac.th (Y.S.); kornkanm@nu.ac.th (K.M.); narisara.p@cmu.ac.th (N.P.); onsaya_k@cmu.ac.th (O.K.); supattar0210@gmail.com (S.Y.); yanping_z@cmu.ac.th (Y.Z.); 2 Department of Community Medicine, Faculty of Medicine, Chiang Mai University, Chiang Mai 50200, Thailand; 3 School of Medical Technology and Artificial Intelligence, Youjiang Medical University for Nationalities, Baise 533000, China

**Keywords:** ferroptosis, iron overload, green tea, *Camellia sinensis*, Huh7, lipid peroxidation, free radicals

## Abstract

Lipid peroxide (LPO) accumulation and a depletion of intracellular antioxidants are hallmarks of ferroptosis, a controlled iron-dependent form of cell death. Iron chelators and radical scavengers can stop it, while erastin or iron overload can cause it. The main catechin in green tea extract (GTE), epigallocatechin-3-gallate (EGCG), has iron-chelating and antioxidant activities. Herein, we investigated the effects of EGCG-rich GTE on ferroptosis in iron-loaded hepatocytes. The contents of EGCG, total phenolics (TPC), and flavonoids (TFC), as well as ABTS^•+^-scavenging activity and cytotoxicity, were determined. Human hepatoma (Huh7) cells were treated with ferric ammonium citrate (FAC) to induce ferroptosis and were co-treated with various concentrations of GTE. Labile iron pool (LIP), reactive oxygen species (ROS), LPO, glutathione (GSH), and glutathione peroxidase 4 (GPX-4) activity were then measured in the cells. One gram of GTE contained 26 mg of EGCG, with a TPC of 172.2 mg gallic acid equivalents and a TFC of 32.9 mg quercetin equivalents. GTE displayed concentration-dependent ABTS^•+^-scavenging activity (IC_50_ = 1.03 mg) that was equivalent to 0.29 mg of Trolox, reporting a Trolox-equivalent antioxidant capacity (TEAC) value of 0.29 mg. High-dose GTE (>100 µM EGCG equivalent) reduced cell viability below 80% (*p* < 0.05). Intracellular LIP, ROS, and LPO levels were markedly elevated, whereas GSH and GPX-4 activity levels were decreased (*p* < 0.05) in iron-loaded Huh7 cells. GTE treatment mitigated these alterations in a dose-dependent manner (*p* < 0.05). These cell-based in vitro findings indicate that EGCG-rich GTE can attenuate ferroptosis-associated oxidative stress in hepatocytes under iron-loading conditions. GTE may serve as a potential dietary antioxidant candidate; further mechanistic studies and in vivo experiments are required to determine its physiological relevance and translational applicability.

## 1. Introduction

Green tea extract (GTE) is abundant with certain polyphenolic catechins, particularly epigallocatechin-3-gallate (EGCG) [1]. EGCG exhibits antioxidant, iron-chelating, anti-inflammatory, and anti-lipid peroxidation properties [2,3]. EGCG’s 3′,4′-dihydroxy and galloyl groups enable it to scavenge free radicals, chelate redox-active iron, and support cellular antioxidant systems [4]. Previous studies have shown that EGCG enhances hepatic antioxidant defense systems by increasing the activity of GPX, glutathione reductase (GR), superoxide dismutase, and catalase activity [5], while decreasing lipid-peroxidation markers, such as 4-hydroxynonenal (4-HNE) and malondialdehyde (MDA) [6]. In animal models of iron overload, EGCG reduces hepatic iron accumulation, improves liver functions, and attenuates oxidative stress [4,7,8], mitigates ferroptosis in pancreatic β-cells and liver tissue [9], and limits steatosis and fibrosis [10]. Moreover, EGCG activates translocation of nuclear factor erythroid 2-related factor 2 (NRF-2) signaling and upregulates GPX-4 and solute carrier family 7 member 11 (SLC7A11) genes, thereby enhancing antioxidant responses under oxidative stress [11,12,13].

Although the antioxidant and iron-chelating activities of EGCG have been well established, its specific ability to modulate ferroptotic pathways in hepatocytes under conditions of iron overload remains insufficiently understood. Existing studies have primarily addressed oxidative stress or apoptosis but have seldom explored specific ferroptotic markers such as labile iron pool (LIP), LPO, GSH depletion, or GPX-4 inactivation. Therefore, the present study aimed to clarify whether EGCG-rich GTE could directly attenuate ferroptosis in iron-loaded human hepatoma (Huh7) cells by integrating the biochemical profiling of GTE with functional ferroptosis assay, thereby addressing a key knowledge gap in the current literature.

## 2. Materials and Methods

### 2.1. Chemicals and Reagents

Aluminum chloride anhydrous powder (Product number 563919, 99.99% pure), L-ascorbic acid (AA) (Product number 255564, ≥99% pure), bovine serum albumin (BSA) (Product number A7030, >96% pure), 3,4,5-trihydroxybenzoic acid or gallic acid (GA) (Product number G7384, 97.5–102.5% pure), 2,2-azino-*bis*-(3-methylbenzothiazoline-6-sulfonic acid (ABTS) (Product number A1888, >98% pure), 6-hydroxy-2,5,7,8-tetramethylchroman-2-carboxylic acid (Trolox) (Product number 238813, 97% pure), 3-(4,5-dimethylthiazol-2-yl)-2,5-diphenyltetrazolium bromide (MTT) (Product number 475989), ERA (Product number E7781, 98% pure), EGCG (Product number E4143, ≥95% pure), catechin (C) (Product number C1251, ≥98% pure), epicatechin (EC) (Product number E1753, ≥90% pure), epicatechin 3-gallate (ECG) (Product number E3893, ≥95% pure), epigallocatechin (EGC) (Product number E3768, ≥95% pure), gallocatechin gallate (GCG) (Product number G6657, ≥98% pure), caffeine (CF) (Product number C1778, ≥95% pure), quercetin (Product number Q4951, 95% pure), Fer-1 (Product number SML0583, ≥95% pure), ferric ammonium citrate (FAC) (Product number F5879), fetal bovine serum (FBS) (Product number F4135), Folin–Ciocalteu reagent (Product number F9252), Bradford’s protein assay reagent (Product number B6916), meta-phosphoric acid (MPA) (Product number 30417-M, ≥85% pure), ortho-phosphoric acid (Product number 695017, >85% in H_2_O), *N*-acetylcysteine (NAC) (Product number A7250, ≥99% pure), potassium acetate (Product number 236497, ≥99% pure), sodium carbonate (Product number S2127, ≥99.5% pure), and thiobarbituric acid (TBA) (Product number T5500, ≥98% pure) were obtained from Sigma-Aldrich Chemical Company, Saint Louis, MO, USA. Dulbecco’s modified Eagle medium (DMEM) (Catalog number 11965092), hydroxyethyl piperazine ethane sulfonic acid (HEPES) (Catalog number A14777, 99% pure), phosphate buffer pH 7.0 (PB) (Catalog number 258595000), and phosphate-buffered saline pH 7.0 (PBS) (Catalog number AM9625) were purchased from Thermo Fisher Scientific, Middlesex Counter, MA, USA. Furthermore, 2′,7′-dichlorohydrofluorescein diacetate (DCFH-DA) (Product code R252-10), *N*-(4-diphenylphosphinophenyl)-*N*′-(3,6,9,12-tetraoxatridecyl)perylene-3,4,9,10-tetracarboxydiimide (Liperfluo) (Product code L248), and FerroOrange (FO) (Product code F374) were purchased from Dojindo Laboratories, Kamimashiki-gun, Kumamoto, Japan. Potassium persulfate (Product number 216224) was purchased from Merck KGaA, Darmstadt, Germany. Ellman’s reagent containing 5,5-dithio-bis (2-nitrobenzoic acid) (DTNB) for colorimetric GSH assay (Catalogue number E-BC-K030-S) and GPX-4 activity assay kit (Catalogue number E-BC-K096-S) were obtained from Wuhan Elabscience Biotechnology Company Limited, Wuhan, Hubei, China. Penicillin and streptomycin were purchased from a drug store in Maharaj Nakorn Chiang Mai Hospital, Faculty of Medicine, Chiang Mai University, Chiang Mai, Thailand. Deionized water (DI), with a density of 0.9985 g/L and a conductivity value of ≤ 4.3 µS/cm, 2,2-azobis-[2-(2-imidazolin-2-yl)propane]dihydrochloride (AIPH) (Product number APOH98737C9A, 98% pure), as well as dimethyl sulfoxide (DMSO) (Product number 102952), were purchased from Merck-Millipore Group, Merck KGaA, Darmstadt, Germany. All organic solvents were of the highest pure HPLC grade. The commercial assay reagents and kits were used according to the manufacturer’s protocol and instructions.

### 2.2. Preparation of Green Tea Extract

GTE was prepared using our established method [7]. Firstly, tea (*Camellia sinensis* var. Siamese) leaf shoots were freshly harvested from an open tea field located at Mon-ngao, Maetaeng District, Chiang Mai, Thailand, in November of 2022. They were immediately dried in a microwave oven (Electric power 800 W) for 3 min at 120 °C to inactivate any inherent polyphenol oxidase and were then ground with a blender to pass through a nylon 60-mesh sieve (NESGTEX, W-TECH Company Limited, Bangkok, Thailand) to obtain a 250 μm particle size. Tea powder (50 g) was extracted with 500 mL of hot deionized water (DI) at 80 °C for 10 min, filtered through cellulose paper (Whatman Company, Maidstone, Kent, UK), and dried using a bench-top vacuum lyophilizer machine (Model FD-10-MR Mini Freeze-Dryer, Labfreez Instruments & Antolab Group Company Limited, Changsha, Hunan, China). Lastly, the lyophilized GTE was kept in a polypropylene bottle with a sealed cap in a 4 °C refrigerator.

### 2.3. HPLC-DAD Analysis of Catechins and Caffeine

Catechin derivatives in GTE were analyzed using the isocratic elution HPLC method established by Wanh et al. [14]. Briefly, GTE was reconstituted in methanol (1.3 mg in 1 mL) and filtered through a syringe membrane filter (polytetrafluoroethylene, PTFE, type, 0.45-μm pore diameter, 13 mm filter diameter, Monotaro Company, Limited, Tokyo, Japan). The GTE and working standard solutions (10 μL) were injected into the HPLC system (Model 1290 Infinity II, Agilent Technologies, Inc., Santa Clara, CA, USA), fractionated on an analytical column (C18 type 150 mm × 4.6 mm, 5 µm particle size, Agilent Technologies, Inc., Santa Clara, CA, USA), capped with a guard column (C18 type 10 mm × 4.6 mm, 5 µm particle size, Agilent Technologies, Inc., Santa Clara, CA, USA) operated at 30 °C, eluted with a mobile-phase solvent (methanol/H_2_O/ortho-phosphoric acid, 20:79.9:0.1 by volume) at a flow rate of 1.0 mL/min, and catechins were then detected with a diode array detector (DAD) at a wavelength of 210 nm. A standard graph for each compound was prepared by plotting the concentration versus peak area (PA). Finally, C, EC, ECG, EGC, EGCG, GA, GCG, and CF were identified by comparing the specific retention time (T_R_) with those of the authentic standards and determining the optimal concentration from the standard graphs. In the analysis, limits of the detection for GA, GCG, EGC, C, EGCG, ECG, and CF were 1.60, 2.28, 1.37, 2.21, 8.31, 0.55, and 20.36 μg, respectively.

### 2.4. Determination of Total Phenolic Content

GTE (100 µL) was mixed with 10% (*v*/*v*) Folin–Ciocalteu reagent (200 µL) and 10% (*w*/*v*) sodium carbonate (800 µL) and incubated at 25 °C for 30 min, and the optical density was measured (OD) at a wavelength of 700 nm against a reagent blank using a double-beam spectrophotometer(Shimadzu Corporation, Nakagyo-ku, Kyoto, Japan). The total phenolic content (TPC) was determined from a standard curve of GA, which was then expressed as mg gallic acid equivalent (GAE)/g [15].

### 2.5. Total Flavonoid Content

GTE (250 µL) was mixed with the chromogenic reagent containing 10% (*w*/*v*) aluminum chloride (50 µL), 1 M potassium acetate (50 µL), and DI (2.15 mL). It was then incubated in the dark at 25 °C for 30 min, and the OD was measured at a wavelength of 415 nm against a reagent blank [16]. Standard quercetin was dissolved in methanol to prepare a standard curve. The total flavonoid content (TFC) was then expressed as mg quercetin equivalent/g (mg QE/g).

### 2.6. Assay of Antioxidant Activity

The total antioxidant activity of GTE was assayed using the ABTS radical cation (ABTS^●+^) decolorization assay, as described by Pelligrini et al. [17]. Initially, the stock blue green cationic ABTS radical (ABTS^•+^) solution was obtained by oxidation of 7 mM ABTS with 2.45 mM (final) potassium persulfate. Then, the stock solution was diluted with PBS, pH 7.4, solution to a concentration providing OD of 0.7 at the wavelength of 734 nm. In the assay, GTE (0.16–2.5 mg EGCG equivalent) or the standard Trolox (water-soluble vitamin E analog) (0.05–0.8 mg/mL) solution (20 µL each) was mixed gently with the ABTS^•+^ solution (1.0 mL). The mixtures were then incubated for exactly 6 min at room temperature, and the OD was measured at a wavelength of 414 nm against the reagent blank.

### 2.7. Cytotoxicity Test

Cytotoxicity profiles of ERA, Fer-1, and FAC were assessed to establish the non-toxic working concentrations for method development and assay optimization. These reagents are commonly used ferroptosis modulators, and determining their tolerated ranges ensured that the subsequent experiments were conducted at concentrations that would not confound the results through off-target cytotoxicity. The cell viability was assayed using a colorimetric MTT test based on mitochondrial reducing enzyme systems that chemically reduce the tetrazolium dye MTT to its insoluble purple color formazan product, thereby reflecting the presence of viable cells [18]. The immortalized Huh7 cell line was kindly provided by Professor John B. Porter, FRCP, FRCPath, MD., Department of Haematology, University College London Medical School, London, United Kingdom. In the assay, Huh7 cells were cultured in DMEM at 37 °C for 12 h and treated with PBS, DFP (25–400 µM), GTE (6.25–100 μM EGCG equivalent), Fer-1 (1.25–20 μM), ERA (0.125–2 μM), and FAC (6.25–400 μM) in a humidified 5% CO_2_ incubator at 37 °C for 24 and 48 h [19]. Then, the cells were incubated with 10 μL of MTT (5 mg/mL) at room temperature for 4 h and centrifuged. Afterward, 200 μL of 1% DMSO was added to the cell pellets. Subsequently, they were resuspended via pipetting and centrifuged. Finally, the supernatant was removed, and the OD value was measured at 570 nm using a 96-well microplate reader (Synergy H4, BioTek Instruments, Winooski, VT, USA) [20].

### 2.8. Investigation of Liver Ferroptosis

#### 2.8.1. Hepatocyte Culture and Iron Loading

The culture conditions and iron-loading procedure were based on previously validated in vitro ferroptosis models. Briefly, FAC is commonly used to elevate intracellular labile iron levels and induce ferroptotic susceptibility in hepatocytes and cancer-derived hepatic cell lines. The concentrations and exposure durations used here follow established protocols demonstrating reproducible intracellular iron accumulation and oxidative stress induction. Under normal conditions, HuH7 cells were cultured in DMEM supplemented with 10% (*v*/*v*) FBS containing 20 μg/dL iron (SI), 69 μg/dL total iron-binding capacity (TIBC), 29% transferrin saturation (TS), penicillin (100 IU/mL), and streptomycin (100 μg/mL) in a humidified 5% CO_2_ incubator at 37 °C, as previously described by Rainey et al. [21] with slight modifications. In addition, 10% FBS was treated with 3 mM FAC (at a final concentration of 300 μM) at room temperature overnight, producing iron-loaded FBS with an SI of 4.11 μg/dL, TIBC of 4.13 μg/dL, and TS of 99.59%. During the course of iron loading, Huh7 cells were incubated with DMEM mixed with the iron-saturated FBS (called DMEM-FAC) for the indicated time, and the cell viability was determined using the MTT assay [19]. Cells with a viability of >80% were used in our experiments.

#### 2.8.2. Treatment of Iron-Loaded Huh7 Cells

The regimen for DFP and GTE followed previously described antioxidant and iron-chelating protocols [22,23]. DFP concentrations similar to those used here have been shown to effectively reduce labile iron and attenuate iron-induced oxidative injury in hepatic cell models. The selected GTE concentrations were based on reported cytoprotective ranges for EGCG–rich preparations. Huh7 cells were treated with PBS, DFP (50 μM), and GTE (3.12–12.5 μM EGCG equivalent) and then cultured in DMEM-FAC in a 5% CO_2_ incubator at 37 °C for 48 h. The treated cells were subsequently centrifuged at 1000× *g* at 4 °C for 10 min using a centrifuge (Type EBA200, fixed-angle rotor, Andreas Hettich GmbH Company, Tuttlingen, Germany), washed twice with PBS, and LIP, ROS, LPO, GSH, and GPX-4 were measured [22]. All the assays were performed in three independent biological replicates, according to the validated methods described below.

#### 2.8.3. Assay of Cellular LIP

FerroOrange is a novel fluorescent probe without chelating properties and used in ferroptosis research, which can react with redox-active Fe^2+^ irreversibly and enable live-cell fluorescent imaging of intracellular Fe^2+^. This assay is sensitive, reproducible, and suitable for high-throughput plate-reader analysis. The redox-active LIP levels were quantified using the fluorogenic FerroOrange probe method, according to Mei et al. [24]. In the assay, FO (1 mM) was diluted in 50 mM HEPES buffer at a pH of 7.4. Subsequently, 2 μL of working FO solution was added to probe the redox-active iron of the treated cells at 37 °C for 30 min. Finally, the fluorescence intensity (FI) was measured at wavelengths of λ_ex_ 543 nm and λ_em_ 580 nm using a 96-well plate spectrofluorometer (Synergy H4, BioTek Instruments, Winooski, VT, USA).

#### 2.8.4. Measurement of Cellular ROS

A non-fluorescent dye H_2_DCFDA is hydrolyzed by intracellular esterases to produce H_2_DCF, which via the probe will be oxidized by ROS to fluorescent DCF, providing a reliable measure of oxidative stress in hepatocytes. Intracellular ROS generation was quantified using the H_2_DCFDA assay previously described by Amer et al. [25], with minor modifications. Briefly, the treated cells were incubated with 9 μM H_2_DCFDA solution at 37 °C for 30 min in the dark, and the green FI of oxidized dichlorofluorescein (DCF) was measured at λ_ex_ = 485 nm and λ_em_ = 525 nm using a 96-well plate spectrofluorometer (Synergy H4, BioTek Instruments, Winooski, VT, USA).

#### 2.8.5. Analysis of Membrane LPO

Liperfluo is a specific fluorescent probe widely used for detecting lipid hydroperoxides, a core biochemical hallmark of ferroptosis. Its sensitivity and selectivity make it a preferred method for assessing LPO in live cells. LPO was determined using Liperfluo fluorochrome, as described by Zheng et al. [26]. Briefly, the treated cells (0.1 mL) were stained with 5 μL of 20 μM Liperfluo solution that had been previously dissolved in 1% DMSO solution and incubated at 37 °C for 15 min. After that, 100 μM cumene hydroperoxide solution was added to the cells and incubated at 37 °C for 2 h. Finally, the cells were washed twice with PBS, and the FI values were measured at λ_ex_ 488 nm and λ_em_ 535 nm using a CytoFLEX flow cytometer (Beckman Coulter Life Sciences, Indianapolis, IN, USA) equipped with 488 nm (blue), 561 nm (yellow green), and (638 nm red) lasers. Data were acquired with Beckman Coulter CytExpert Version 2.6 software for Acquisition and Analysis and analyzed in FlowJo version 11 installed with CytExpert software. Acquisition was run at a low flow rate (~10–20 µL/min) to minimize coincident events. Photomultiplier tube voltages/gains were optimized using unstained and single-stained controls and kept constant within each experiment. For each sample, we collected live singlet events after applying the gating strategy. To maintain parity across samples, acquisition was stopped at 10,000 viable singlets per sample (or 20,000 when parity was not required).

#### 2.8.6. Determination of Cellular GSH Content

GSH levels were estimated with a classical spectrophotometric method in redox biochemistry using Ellman’s reagent (DTNB) following the method of Moron et al. [27]. The DTNB–GSH reaction generates the chromophore TNB, allowing sensitive measurement of intracellular GSH levels. The treated cells were lysed with hypotonic PB solution. The cell lysate was then deproteinized with 25% trichloroacetic acid and centrifuged at 12,000 rpm (6900× *g*) at 4 °C for 10 min. Afterward, the supernatant was collected and incubated with Ellman’s reagent containing 0.2 M PB at pH values of 8.0 and 0.06 mM DTNB for 10 min at room temperature. Finally, the OD value of the colored product was measured at 412 nm against a reagent blank using a UV/visible double-beam spectrophotometer (Shimadzu Corporation, Nakagyo-ku, Kyoto, Japan). The measured GSH content was the normalized total protein concentration determined by the Bradford method, as described below.

#### 2.8.7. Assay of GPX-4 Activity

The coupled enzyme assay used here is derived from established GPX-4 activity measurement protocols, in which NADPH consumption is monitored in the presence of glutathione reductase and excess GSH. This method is commonly employed for detecting ferroptosis-related decreases in GPX-4 enzymatic function. GPX-4 activity was measured using a coupled enzyme assay, as described by Flohé and Günzler [28]. In principle, GPX-4 catalyzes the conversion of H_2_O_2_ and GSH substrates to H_2_O and oxidized glutathione (GSSG) products, wherein the GSSG will consume NADPH_2_ with the addition of GR. In analysis, the cell lysate was diluted 1:1 (*v*/*v*) with 0.9% NaCl/stabilizer solution and centrifuged at 12,000 rpm (6900× *g*) at 4 °C for 10 min. Next, 20 µL of the clear supernatant was added to each of the sample and control wells. The reaction solution was then added to the sample well, and the inhibitor solution (40 μL each) was added to the control well. After five seconds of full mixing, they were allowed to sit at room temperature for fifteen minutes. Lastly, each well received 40 μL of the working GR solution. Using a BioTek microplate reader, the OD values of NADPH were promptly measured at 340 nm and noted. Consequently, GPX-4’s specific activity (units/mg of protein) was calculated using Formula (1):

(1) Specific activity = (∆OD_sample_ − ∆OD_control_) ÷ (ε × d) × (V_total_ ÷ V_sample_) ÷ T × 2 ÷ C_protein_,

where ∆OD_sample_ represents the change in the OD value of the sample well; ∆OD_control_ represents the change in the OD value of the control well; the molecular extinction coefficient (ε) is defined as the molar extinction coefficient of the product at 340 nm, which is 6.22 × 10^−3^ L/μmol/cm; d signifies the optical path of the solution height, measured at 0.6 cm; V_total_ indicates the total volume of the reaction, which is 0.24 mL; V_sample_ refers to the volume of the sample, which is 0.02 mL; T represents the duration of the lysate sample reaction, set at 15 min; C_protein_ is the concentration of protein in the lysate sample, expressed in g/L; and 2 is the dilution factor applied to the sample prior to testing. Consequently, the quality control metrics of the assay kit revealed a sensitivity value of 3.22 U/L, a detection range of 3.22–44.69 U/L, an inter-assay coefficient of variation (CV) ranging from 0.4% to 3.4%, and an intra-assay CV ranging from 1.2% to 3.1%. The measured GPX-4 activity was the normalized total protein concentration determined by the Bradford method, as described below.

#### 2.8.8. Measurement of Protein Content

The Bradford’s dye-binding assay is a widely used colorimetric method for protein quantification based on Coomassie Brilliant Blue G-250 dye binding. Its rapidity, compatibility with cellular lysates, and minimal interference make it suitable for normalizing biochemical assays in cultured cell studies [29]. In the assay, 100 µL of cell lysate and BSA standard were incubated with 1.0 mL of Coomassie-brilliant blue reagent at room temperature for 5 min, and the OD value of the product was measured at a wavelength of 595 nm against a reagent blank. The amount of protein was determined based on a calibration curve prepared from the BSA solution (0.125–2 mg/mL).

#### 2.8.9. Identifying Phenolics and Their Metabolites with HPLC-MS

The FAC-loaded Huh7 cells were treated with EGCG (25 μM) and GTE (25 μM EGCG equivalent) for 24 h; then, a slightly modified version of the HPLC-MS method was used for identification of phenolics and their metabolites [30,31]. Our setup was based on an Agilent 1100 Series HPLC system (Agilent Technologies 1100 Series, Deutschland GmbH, Waldbronn, Germany) equipped with a quaternary pump (G1311A), online vacuum degasser (G1322A), autosampler (G1313A), thermo-stated column compartment (G1316A), and a PDA detector (G1315A). The HPLC was connected to an Agilent 1100 LC/MSD SL mass spectrometer with an atmospheric pressure ESI interface, using a 1:1 flow splitter. Before analysis, we prepared each sample by dissolving C and EGCG standard (5 mg each) and the extract in 1.0 mL of a 1:1 mixture of acetonitrile (solvent A) and 10 mM formate buffer at pH 4.0 (solvent B). We then filtered the solutions through a 0.45 µm PTFE syringe filter. We injected a 20 μL aliquot of the samples onto a LiChroCART RP-18e column (150 mm × 4.6 mm, 5 µm particle size). The column was kept at a steady 40 °C. Mobile phases A and B were pumped at a flow rate of 1.0 mL/min using the following gradient: the run started with 100% B for 5 min and then ramped to 20% A from 5 to 10 min. This 20% A concentration was held for 10 min, followed by an increase to 40% A over the next 40 min. After holding at 40% A for 3 min, the system returned to the initial 100% B condition for 5 min to re-equilibrate. The eluent was first monitored by the PDA detector at 280 nm. For the mass spectrometry, we used the positive ESI mode and scanned a mass-to-charge ratio (*m*/*z*) range of 100 to 700. The key source parameters were an ESI energy of 70 eV, an ion source temperature of 150 °C, and an interface temperature of 230 °C. We used nitrogen as the nebulizing, drying, and collision gas. The nebulizer pressure was set to 60 psi, and the drying gas flowed at 13 L/min. The capillary voltage was set to 3500 V, and the oven temperature was programmed with an initial hold at 80 °C for 3 min, followed by a ramp to 110 °C at 10 °C/min (held for 5 min), an increase to 190 °C (held for 3 min), a ramp to 220 °C at 10 °C/min (held for 4 min), and a final increase to 280 °C at 15 °C/min (held for 13 min). We used an external standard (Agilent ESI-L Low Concentration Tuning Mix) for automatic mass calibration. Our method achieved a limit of detection (LOD) of 0.5 mg/kg, an LOD of 1.20 mg/kg, and recovery values between 70% and 110%. All data acquisition and analysis, including chemical formula prediction and exact mass calculations, were handled using MassHunter software version B.04.00 (Agilent Technologies, Inc., Santa Clara, CA, USA).

### 2.9. Statistical Analysis

The results and data were obtained from three independent biological replicates, analyzed using the Statistical Package for the Social Sciences Statistics (SPSS) program version 21 for Windows (IBM Corporation, Armonk, NY, USA) and expressed as mean ± standard deviation (SD). All assays, including GPX-4 activity, GSH content, ROS, LPO, and LIP measurements, were conducted using three independent biological batches of cells prepared on different days. For experiments involving multiple groups, one-way analysis of variance (ANOVA) followed by Tukey’s post hoc test was applied to correct for multiple comparisons. Statistical significance was based solely on biological replicates. A *p* value of less than 0.05 was deemed to be statistically significant.

## 3. Results

### 3.1. Chemical Compositions of GTE

**Figure 1:**
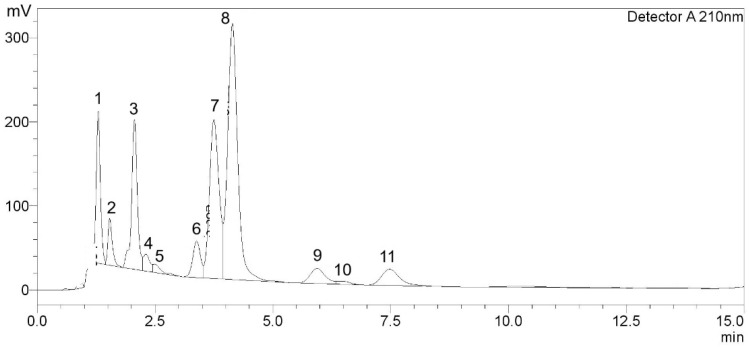
Chromatographic peaks of catechins and caffeine in GTE.

HPLC-DAD analysis of GTE (1.3 mg/mL) revealed 11 chromatographic peaks corresponding to catechins, caffeine, and several unidentified compounds (Figure 1 and Table 1). Among the identified constituents, GA eluted at 1.297 min with a quantified amount of 7.96 ± 0.31 mg/g, GCG appeared at 1.537 min (3.36 ± 0.20 mg/g), followed by EC at 3.385 min (1.24 ± 0.02 mg/g), and ECG at 7.478 min (2.50 ± 0.01 mg/g). The major catechin, EGCG, exhibited a prominent peak at 3.750 min and was present at 8.38 ± 0.18 mg/g. A substantial peak corresponding to CF eluted at 4.142 min, with a concentration of 31.89 ± 0.51 mg/g. Thus, EGCG accounted for approximately 54.13% of the total quantified catechins (GCG, EC, EGCG, and ECG). Several additional peaks were detected throughout the chromatogram; nonetheless, they were identified or below the limit of detection.

In the chemical analysis, 1 g of GTE contained 172.2 ± 1.6 mg GAE for TPC and 32.9 ± 0.7 mg QE for TFC.

### 3.2. Antioxidant Activity of GTE

**Figure 2:**
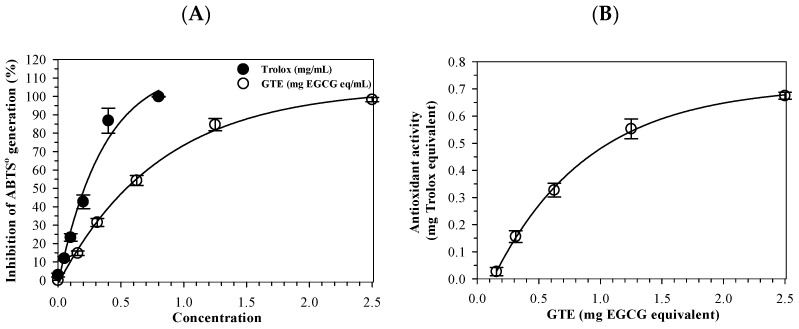
Antioxidant activity of green tea (GTE) and Trolox determined by the ABTS^•+^-scavenging assay. Data are presented as mean ± SD of three independent experiments. Abbreviations: ABTS^•+^ = 2,2-azino-*bis*-(3-methylbenzothiazoline-6-sulfonic acid cationic radical, EGCG = epigallocatechin 3-gallate, GTE = green tea extract.

The ABTS^+^ radical-scavenging assay demonstrated that both GTE (0.16–1.25 mg EGCG equivalent) and standard Trolox (0.05–0.8 mg/mL) inhibited ABTS^•+^ generation in a concentration-dependent manner. (Figure 2A). As the concentration increased, the radical-scavenging activity rose proportionally for both agents, confirming that the assay responded linearly within the tested range. Accordingly, the half-maximal inhibitory concentration (IC_50_) value of GTE was recorded at 1.03 mg and compared to that of Trolox at 0.29 mg, showing that while Trolox is the more potent antioxidant on a per mass basis, GTE still has substantial activity attributable to its catechin content, particularly EGCG. In addition, the Trolox equivalent antioxidant capacity (TEAC) is expressed as µg Trolox equivalents per g GTE (Figure 2B). Quantitatively, 1 g of GTE showed an antioxidant capacity equivalent to 495 ± 52 mg of Trolox, indicating GTE possesses strong electron-donating and radical-quenching ability. These findings validate the extract’s chemical antioxidant potential and provide a mechanistic foundation for its protective effects against ferroptosis observed in later experiments.

### 3.3. Cytotoxicity of GTE

**Figure 3:**
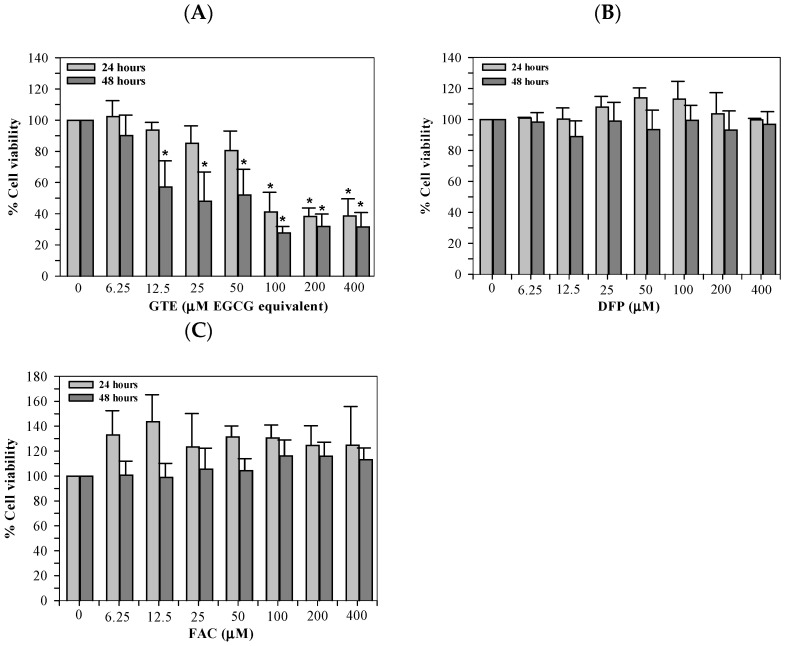
Viability of Huh7 cells cultured in DMEM and treated with GTE (**A**), DFP (**B**), and FAC (**C**) for 24 and 48 h. Data are presented as mean ± SD of three independent experiments. Accordingly, * *p* < 0.05 indicates a significant difference compared with untreated control cells. Abbreviations: DFP = deferiprone, DMEM = Dulbecco’s modified Eagle medium, EGCG = epigallocatechin 3-gallate, FAC = ferric ammonium citrate, and GTE = green tea extract.

As part of the preliminary assay optimization, we evaluated the toxicity of ERA, Fer-1, and FAC to identify the concentration ranges that were compatible with Huh7 cell survival. Although ERA and Fer-1 were not included in the final ferroptosis experiments, these data confirmed their safety margins and prevented the use of cytotoxic concentrations during pilot testing. GTE was determined to be toxic to Huh7 cells in concentration-dependent manners, with significant differences found at 100–400 μM EGCG equivalent and 12.5–400 μM EGCG equivalent when incubated for 24 and 48 h, respectively (Figure 3A). However, treatments of DFP (6.25–400 μM), and FAC (6.25–400 μM) were not harmful to the Huh7 cells (Figure 3B and Figure 3C, respectively) when incubated for 24 and 48 h.

### 3.4. Cellular LIP and ROS Content

**Figure 4:**
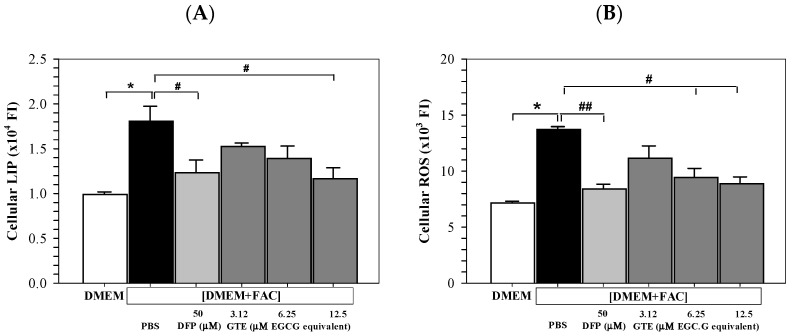
Levels of LIP (**A**) and ROS (**B**) in Huh7 cells cultured in DMEM or PBS [DMEM + FAC] and treated with PBS, DFP (50 μM), and GTE (3.12–12.5 μM EGCG equivalent) for 48 h. Data are presented as mean ± SD obtained of three independent experiments. Accordingly, * *p* < 0.05 versus DMEM control, ^#^ *p* < 0.05, ^##^ *p* < 0.01 versus PBS [DMEM + FAC]. Abbreviations: DFP = deferiprone, DMEM = Dulbecco’s modified Eagle medium, [DMEM − FAC] = ferric ammonium citrate-added DMEM, EGCG = epigallocatechin 3-gallate, GTE = green tea extract, PBS = phosphate-buffered saline.

The iron loading significantly increased the redox-active LIP levels in Huh7 cells. Furthermore, treatments of DFP (50 μM) and GTE (3.12–12.5 μM EGCG equivalent) restored an increase in LIP in iron-loaded Huh7 cells significantly and tentatively, depending upon the GTE dose (Figure 4A). Similarly, ROS levels were increased in Huh7 cells cultured in [DMEM + FAC], and the increased ROS was reinstated significantly by DFP and GTE treatments, depending upon the GTE dose (Figure 4B).

### 3.5. Membrane LPO Content

**Figure 5:**
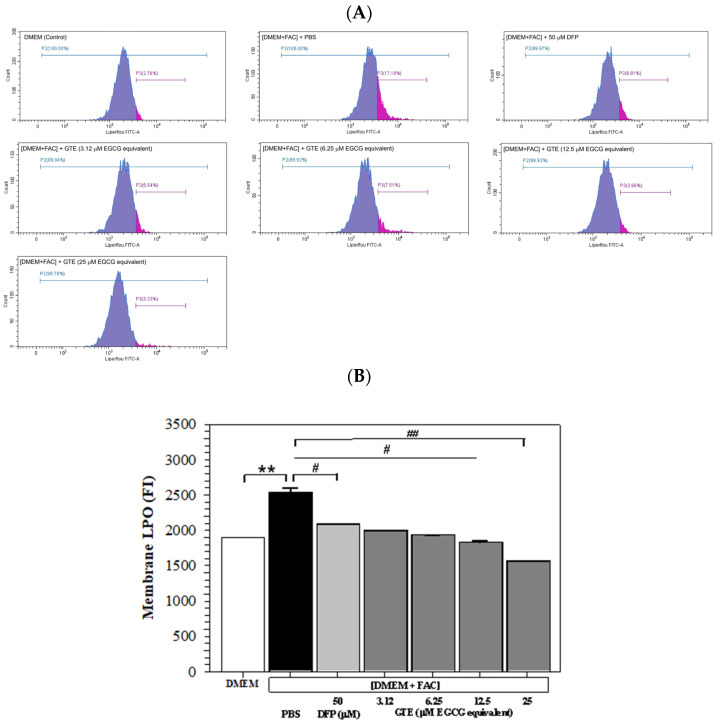
Histogram plots (**A**) and mean ± SD values of three independent experiments (**B**) of LPO accumulation in Huh7 cells incubated with or without 150 μM FAC and treated with PBS, 50 μM DFP, and GTE (3.12, 6.25, 12.5, and 25 μM EGCG equivalent) for 48 h. Accordingly, ** *p* < 0.05 versus DMEM control; ^#^ *p* < 0.05, ^##^ *p* < 0.01 versus PBS [DMEM + FAC]. Abbreviations: DFP = deferiprone, DMEM = Dulbecco’s modified Eagle medium, [DMEM + FAC] = ferric ammonium citrate-added DMEM, EGCG = epigallocatechin 3-gallate, eq = equivalent, FAC = ferric ammonium citrate, FI = fluorescence intensity, GTE = green tea extract, PBS = phosphate-buffered saline.

Histogram plots generated from the flow cytometry analysis depict cells stained with a fluorescence dye and demonstrate the FI signal on the x-axis, along with the number of counts or events on the y-axis. As shown in Figure 5A, the FI signal exhibited a significant elevation in Huh7 cells subjected to incubation with [DMEM + FAC] in contrast to those incubated with DMEM alone. This would indicate an increased membrane LPO level in iron-overloaded Huh7 cells, and the increase was restored by treatments of AA (100 μM) and GTE (3.12–25 μM EGCG equivalent). Quantitatively, LPO was significantly increased in the iron-loaded hepatocytes and reinstated by AA and GTE treatments, which were also dependent upon the dose (Figure 5B).

### 3.6. Cellular GSH

**Figure 6:**
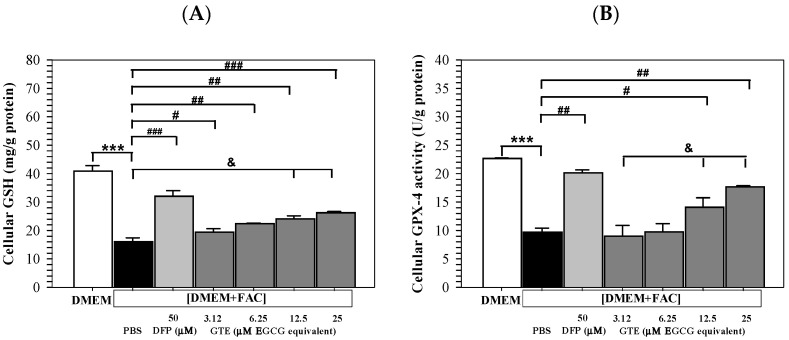
Levels of GSH (**A**) and GPX-4 activity (**B**) in Huh7 cells incubated in DMEM or [DMEM + FAC] and treated with PBS, DFP (50 μM), and GTE (3.12–25 μM EGCG equivalent) for 48 h. Data are presented as mean ± SD of three independent experiments. Accordingly; *** *p* < 0.005 versus DMEM control; ^#^ *p* < 0.05, ^##^ *p* < 0.01, ^###^ *p* < 0.005 versus PBS [DMEM + FAC]; ^&^ *p* < 0.05 between GTE treatment groups. Abbreviations: DFP = deferiprone, DMEM = Dulbecco’s modified Eagle medium, [DMEM + FAC] = ferric ammonium citrate-added DMEM, EGCG = epigallocatechin 3-gallate, GPX-4 = glutathione peroxidase 4, GSH = reduced glutathione, GTE = green tea extract, PBS = phosphate-buffered saline.

Remarkably, iron loading by [DMEM + FAC] addition significantly decreased the GSH levels in Huh7 cells cultured in DMEM. At the same time, treatments of DFP (50 μM) and GTE (3.12–25 μM EGCG equivalent) significantly reinstated the increased GSH contents in a dose-dependent manner (Figure 6A). Consistently, GPX-4 activity was significantly decreased in Huh7 cells subjected to incubation with [DMEM + FAC] in contrast to those incubated in DMEM alone. The activity was significantly restored after treatments of DFP (50 μM) and GTE (3.12–25 μM EGCG equivalent), depending upon the GTE dose (Figure 6B).

### 3.7. Catechins and Their Metabolites in Culture Medium and Cell Lysate

**Figure 7:**
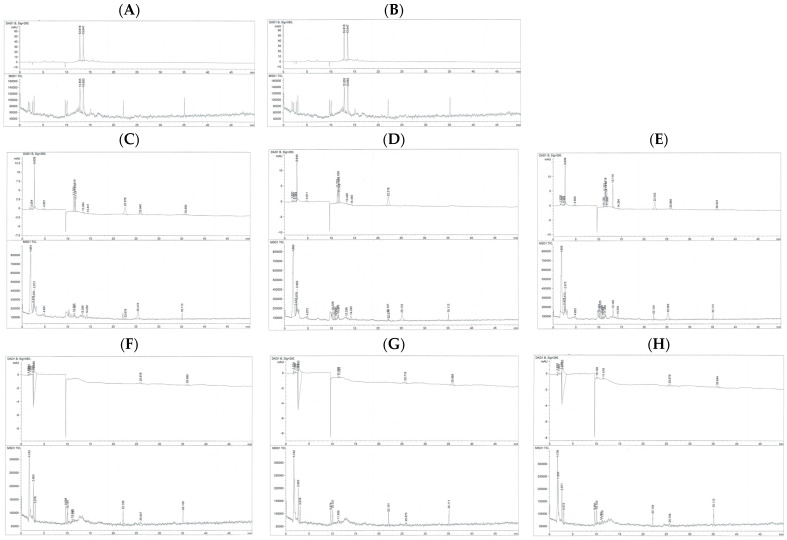
Chromatograms of different phenolic substances obtained by the HPLC-DAD–MS: (**A**) = standard catechin, (**B**) = standard EGCG, (**C**) = medium (no treatment), (**D**) = medium (EGCG treatment), (**E**) = medium (GTE treatment), (**F**) = cell lysate (no treatment), (**G**) = cell lysate (EGCG treatment) and (**H**) = cell lysate (GTE treatment).

The chromatograms showed that the catechin standard eluted as two closely spaced stereoisomeric peaks (catechin and epicatechin) at approximately 12.86 min (Figure 7A), and its MS spectrum was dominated by the expected *m*/*z* 291 ion (Figure S1A), confirming the compound’s identity. In contrast, the EGCG standard displayed a single well-resolved chromatographic peak at 13.58 min (Figure 7B), and its MS spectrum displayed the parent ion *m*/*z* 459.0 [M+H]^+^, along with the dominant fragment at *m*/*z* 291.0 generated by loss of the gallate moiety; several additional lower-intensity fragments (*m*/*z* 313, 336, and 351) and characteristic small ions (*m*/*z* 100.2, 115.1, 126.2, 139.1, 152.1, 166.2, 178.1, 194.1, 204.2, 224.2, and 233.9) (Figure S1B) further confirmed EGCG identity and purity. In the untreated Huh7 cell lysate, the chromatogram showed no distinct small-molecule peaks (Figure 7C), and the MS spectra were dominated by broad low-intensity ions between *m*/*z* 100 and 300, with occasional higher-mass ions (*m*/*z* 500–650) typical of endogenous cellular background (Figure S1C). Importantly, no catechin- nor EGCG-related ions (*m*/*z* 291 or 459) were observed, establishing this sample as the baseline negative control. Similarly, in the EGCG-treated cell lysate, the chromatogram lacked a peak at the EGCG retention time (13.6 min) (Figure 7D), and the MS spectra did not contain the diagnostic *m*/*z* 459 [M+H]^+^ or *m*/*z* 291 ions (Figure S1D), indicating that no EGCG was detectable in the cells. The GTE-treated lysate likewise showed no detectable catechins, EGCG, or their metabolites, with chromatograms lacking peaks at expected retention times (Figure 7E) and MS spectra lacking their parent or fragment ions (Figure S1E).

The culture medium from untreated cells contained only background signals and no peaks eluting at 12–14 min (Figure 7F), and the MS spectra showed no catechin- or EGCG-related ions (*m*/*z* 289–291 or 459), confirming the absence of polyphenols or metabolites (Figure S1F). The medium from EGCG-treated cells similarly showed no detectable EGCG or EGCG-derived metabolites, with no chromatographic peaks corresponding to catechin (12.8 min), epicatechin (12.8–13.0 min), or EGCG (13.6 min) (Figure 7G,H). Consistently, no parent ions for catechin/epicatechin (*m*/*z* 289–291) or EGCG (*m*/*z* 459 [M+H]^+^), nor their characteristic fragment ions (*m*/*z* 291, 273, 247, 219, etc.), were detected (Figure S1G,H). Overall, across all Huh7 cell experiments, neither the cells nor the culture media contained detectable levels of catechin, EGCG, or their common metabolites under the conditions tested. Due to the inability to detect parent catechins, EGCG, or their metabolites in either lysate or medium, the findings likely reflect a combination of rapid chemical degradation, protein binding, poor cellular uptake, and low intracellular metabolite abundance, rather than analytical insensitivity.

## 4. Discussion

The present findings provide new mechanistic insight by demonstrating that EGCG- and CF-rich GTE suppresses ferroptosis in hepatocytes subjected to iron overload, a mechanism that has not been comprehensively explored in previous studies [1,2,3,4]. The chromatographic analysis of GTE demonstrated that CF and catechins were the principal constituents of the extract. Among these, CF was the most abundant compound, consistent with typical green tea composition. EGCG was the most dominant catechin, showing a strong peak in the chromatogram, while other catechins including GCG, EC, and ECG were present in smaller amounts but remained relevant to the overall polyphenolic profile. The presence of unidentified peaks suggests that additional minor phytochemicals were present in the extract but were not identified or quantified. Consistently, EGCG and other constituents (such as EC, ECG, EGC, and minor flavonols) in green tea may act synergistically to modulate the cellular redox-active iron and any relevant antioxidant defenses [32]. The combined and possibly interactive actions of these polyphenols could potentially contribute to the overall bioactivity of GTE beyond that of isolated EGCG [2]. Metabolomic approaches will therefore be required to delineate the individual and collective roles of these catechins in ferroptosis regulation.

There are several antioxidant activity assays, such as ABTS, 2,2-diphenyl-1-picrylhydrazyl, ferric reducing antioxidant property, and oxygen radical absorbance capacity. The ABTS was chosen because it allows the assessment of both hydrophilic and lipophilic antioxidant compounds, shows good reproducibility, and provides rapid sensitive quantification of radical-scavenging activity over a broad pH range. We acknowledge that chemical antioxidant assays have limited physiological relevance and do not necessarily reflect intracellular redox dynamics. Therefore, the ABTS data were used only to benchmark the extract’s chemical antioxidant potential and not to infer biological mechanisms. Our conclusions are instead supported by cell-based measurements directly relevant to ferroptosis, including ROS production, LIP accumulation, LPO levels, GSH depletion, and GPX-4 activity. These features makes it particularly suitable for complex plant extracts such as GTE, which contain multiple catechins and polyphenolic constituents with varying solubilities.

The toxicity assays for ERA, Fer-1, and FAC were conducted to determine safe non-cytotoxic concentrations for assay optimization. These reagents represent classical ferroptosis modulators, and establishing their viability thresholds ensured that the concentrations selected for iron loading and ferroptosis assessment did not induce off-target cytotoxicity. We recognize that higher concentrations of GTE reduced cell viability in our cytotoxicity assays, indicating potential dose-dependent toxicity. This finding aligns with those of previous studies reporting that concentrated catechins may exert pro-oxidant or cytotoxic effects under certain in vitro conditions. Importantly, the concentrations of GTE that conferred protection against iron-induced ferroptosis were substantially lower than those associated with reduced viability, suggesting a reasonable in vitro therapeutic window. Because in vitro cytotoxicity does not necessarily predict in vivo toxicity—owing to differences in metabolism, distribution, and clearance—the present results should not be over-interpreted with regard to clinical safety. Nevertheless, we acknowledge this limitation and emphasize that future translational studies should further investigate safe dosing ranges and potential hepatotoxicity associated with high catechin exposure.

Our study demonstrates that GTE attenuates multiple hallmark ferroptosis endpoints—including LIP accumulation, ROS generation, LPO, GSH depletion, and reduced GPX-4 activity. Consistently, GTE treatment restored these parameters in a dose-dependent manner, demonstrating both antioxidant and iron-modulating properties consistent with EGCG’s dual function as a free radical scavenger and iron chelator [2,3,33]. EGCG reduced hepatic iron accumulation and upregulated antioxidant defenses, including GPX-4 and NRF-2, in iron-overloaded mice [34]. We acknowledge that these findings are primarily functional and descriptive. Moreover, we did not assess upstream regulatory pathways such as NRF2 activation, SLC7A11 expression, or other molecular mediators that may govern the ferroptosis-protective effects of GTE [34,35,36]. Although previous studies have reported that EGCG can activate NRF2 signaling and modulate antioxidant genes, the present work did not directly evaluate pathway-specific gene or protein expression. Future studies incorporating mechanistic assays such as Western blotting, qPCR, ferroptosis-related inhibitors, or genetic perturbation will be essential to delineate whether GTE exerts its effects through canonical NRF2/SLC7A11/GPX-4 pathways or through alternative mechanisms.

Interestingly, our data also reflect the biphasic nature of EGCG activity: low to moderate doses (3.12–25 μM) were protective against the iron-loaded Huh7 hepatocytes, whereas higher concentrations (>100 μM) reduced cell viability, paralleling previous studies of pro-oxidant effects at supraphysiological catechin levels [2,3]. This observation aligns with the well-recognized hormetic behavior of EGCG: while low-to-moderate concentrations act as antioxidants, high micromolar levels may undergo auto-oxidation, generating H_2_O_2_ and quinone intermediates that confer pro-oxidant and cytotoxic effects in vitro [37,38]. In isolated hepatocyte cultures, 200 µM EGCG induced dose- and time-dependent cytotoxicity that correlated with intracellular ROS accumulation, which could be mitigated by catalase or glutathione supplementation [9,34,36,39]. Consistently, animal studies demonstrate hepatotoxicity following high expose to oral EGCG. CF-1 mice that had been administered with two once-daily doses of 750 mg/kg EGCG had markedly elevated serum alanine aminotransferase (ALT) and liver necrosis. These outcomes were also accompanied by increased oxidative stress and DNA damage markers [3,35,40,41]. A meta-analysis of green tea extract–associated hepatotoxicity identified 27 probable cases of acute liver injury [42], and a randomized controlled trial showed significantly greater ALT elevation among women consuming high-dose GTE when compared with a placebo [43]. Hence, translational applications of GTE should emphasize lower sub-cytotoxic doses that are consistent with achievable exposure levels, incorporate hepatic safety monitoring, and evaluate combination or fractionated dosing strategies to sustain efficacy while minimizing risks. While our findings highlight the anti-ferroptotic potential of GTE, the observed decline in cell viability at high doses underscores the necessity of cautious dose selection in preclinical and clinical contexts.

Using HPLC-DAD-MS, we demonstrated that neither catechin nor EGCG, nor their expected phase I/II metabolites, were detectable in Huh7 cells or culture medium following treatment, despite clear and well-defined signals observed in analytical standards. This outcome is consistent with the known instability of green tea catechins under physiological culture conditions, particularly the rapid oxidation, epimerization, and auto-degradation of EGCG at neutral pH and in the presence of serum proteins [44,45]. Several studies have also shown that EGCG exhibits poor cellular uptake in hepatocyte-derived lines due to limited membrane permeability and extensive extracellular binding [46]. Additionally, once inside cells, catechins are rapidly conjugated to glucuronides, sulfates, and methylated metabolites, often at levels that fall below detection in untargeted MS workflows [47]. Likewise, dimethyl-EGCG (half-life 4.1 h) and EGCG (half-life 2.7 h) were detected in human plasma and urine from rats and mice following green tea ingestion [48]. Moreover, several catechins and their metabolites (e.g., methylcatechins, dimethylcatechins, catechin glucuronide, catechin sulfate) were detected in plasma samples from β-thalassemia patients following green tea consumption [49]. Thus, our inability to detect parent catechins, EGCG, or their metabolites in either lysate or medium likely reflects a combination of rapid chemical degradation, protein binding, poor cellular uptake, and low intracellular metabolite abundance, rather than analytical insensitivity. These findings align with prior reports emphasizing the challenges of quantifying green tea polyphenols in vitro and underscore the need for stabilized formulations, antioxidant additives, or targeted HPLC-MS/MS methods to improve detection in cell-based systems.

We have revealed that GTE reverses multiple ferroptotic markers, including LIP, ROS, LPO, GSH, and GPX-4 within the same experiment window, establishing a mechanistic link between iron overload and hepatocellular ferroptosis attenuation. In addition, we have connected quantified EGCG and antioxidant indices to cellular outcomes, providing direct a chemical-to-biological relationship rarely emphasized in prior green tea studies. Moreover, by using iron-saturated serum [DMEM + FAC] as a physiologically relevant model, we have revealed that GTE modulates the LIP itself rather than merely scavenging ROS, which deepens the mechanistic understanding of catechin-mediated hepatoprotection. Furthermore, while GTE and EGCG exhibit general antioxidant properties, several observations support a ferroptosis-specific mechanism rather than nonspecific ROS scavenging. The concurrent restoration of GPX-4 activity, reduction in lipid peroxidation, and normalization of the labile iron pool are hallmark signatures of ferroptosis suppression. The similarity between the effects of GTE and those of ferroptosis-specific inhibitors such as Fer-1 and DFP further implicates an iron-dependent lipid peroxidation pathway [34,50,51,52,53]. Regarding the stability caveat, EGCG auto-oxidation in culture media can generate H_2_O_2_ and related products that may confound readouts. Although we did not quantify H_2_O_2_ or apply stabilizers in the present experiments, this is a recognized methodological limitation. Future work will document the stability (e.g., light/pH control, as well as ascorbate and metal chelators) and monitor medium oxidants to ensure that the observed effects reflect cellular modulation rather than chemical degradation [54].

In terms of limitations, first, the study employed only one batch of GTE sample, which limits the generalizability and reproducibility of the results. Plant-derived extracts can vary in catechin and other phytochemical compositions and antioxidant capacity depending on strains, geographic altitude, harvest time and season, location, and processing. Hence, employing multiple independent GTE batches is warranted to validate these findings and account for potential natural composition variations. In the absence of an ultrahigh pressure liquid chromatography–quadrupole time-of-flight mass spectrometry (UHPLC-QTOF-MS) instrument in our laboratory, we did not measure the intracellular EGCG or its phase-II conjugates in cell lysates or the culture medium. Consequently, we cannot confirm the cellular exposure profiles or link specific metabolite parent ratios to the observed anti-ferroptotic effects. Our interpretation, therefore, remains at the extract level, with EGCG-equivalent dosing used as a quantitative reference rather than proof that EGCG alone mediates the phenotype. Future research will overcome this restriction by quantifying parent EGCG and conjugate, using targeted UHPLC-QTOF-MS and relating exposure to LIP, ROS, LPO, GSH, and GPX-4 dynamics [55]. We used a single Huh7 hepatocyte line and one iron-loaded paradigm (FAC-saturated FBS), which may not capture the in vivo complexity. Validation in primary hepatocytes or animal models is warranted. Huh7 cells are widely used as an in vitro hepatic model due to their retained metabolic and antioxidant enzyme activities; they are nonetheless cancer-derived and may not fully recapitulate the ferroptotic responses of normal hepatocytes under iron overload. Their altered redox regulation, proliferation rate, and basal iron metabolism could influence the sensitivity to ferroptosis inducers and antioxidants [56]. Therefore, non-tumorigenic hepatocyte models (e.g., HepaRG or primary human hepatocytes) and in vivo iron overload systems should be used to confirm the physiological relevance and strengthen the translational implications of green tea catechins as potential dietary modulators of hepatic ferroptosis [34]. The relative contributions of other polyphenols were not dissected; future use of fractionation of EGCG depletion can clarify this. The concentrations used in vitro may exceed the physiological plasma levels; therefore, pharmacokinetic studies are needed to establish a clinically relevant dosing. Finally, our analyses focused on biochemical markers.

For future directions, studies should confirm whether the observed effects are consistent across multiple extract batches. Lipidomics and the inclusion of ferroptosis-specific inhibitors such as Fer-1 and liprostatin-1 across all endpoints would refine the pathway specificity. Additionally, comparing GTE with classical antioxidants, such as N-acetylcysteine, and assessing ferroptosis marker proteins and genes, like acyl CoA synthetase long chain family member 4 (ACSL4), SLC7A11, arachidonate 15-lipoxygenase, and prostaglandin-endoperoxide synthase 2, would help confirm pathway specificity. Lastly, performing *GPX-4* gene knockdown or RSL3 challenge experiments would clarify whether GTE acts primarily through GPX-4 dependent antioxidant restoration or via upstream iron chelation. Such complementary analyses will enhance the mechanistic resolution and strengthen the translational relevance of GTE as a potential dietary modulator of hepatic ferroptosis.

Taken together, our results reinforce that green tea extract abundant with EGCG and antioxidant constituents attenuate iron-induced ferroptosis in hepatocytes by normalizing the iron redox balance and restoring the GSH/GPX-4 system. This supports the therapeutic potential of GTE as a natural ferroptosis inhibitor for oxidative stress-related liver disorders, complementing prior animal and cellular studies on catechin-mediated hepatoprotection [34,36,39,57,58].

## 5. Conclusions

The findings of our study demonstrate that green tea extract rich in EGCG and caffeine and processing with high phenolic and flavonoid contents effectively mitigates iron-induced ferroptosis in huma hepatoma (Huh7) cells. GTE significantly decreased the LIP, ROS, LPO, GSH, and GPX-4 activity levels. These findings confirm that GTE protects hepatocytes by maintaining redox homeostasis and stabilizing antioxidant defense system. All of this research points to GTE as a naturally occurring ferroptosis inhibitor that may be useful as a treatment for liver diseases associated with iron overload. Future work should include UHPLC-QTOF-MS or HPLC-DAD profiling to determine the relative contribution of individual catechins to ferroptosis inhibition. Using primary hepatocytes and in vivo models are warranted to validate these effects, define the bioavailable dose ranges, and elucidate the molecular target, paving the way for translational development of green tea-derived formulations in oxidative-associated liver disorders.

## Figures and Tables

**Table 1:** Amounts of catechins and CF in GTE. Data obtained from two separate analyses are expressed as mean ± SD.

Peak	T_R_ (min)	PA	PH	Compounds	Amount (mg/g)
1	1.297	1,128,089	180,566	GA	7.96 ± 0.31
2	1.537	358,332	55,712	GCG	3.36 ± 0.20
3	2.064	1,494,302	177,913	Unknown	-
4	2.310	198,745	20,126	C	ND
5	2.495	101,230	9911	Unknown	-
6	3.385	490,797	43,292	EC	1.24 ± 0.02
7	3.750	2,704,511	188,897	EGCG	8.38 ± 0.18
8	4.142	4,523,257	304,418	CF	31.89 ± 0.51
9	5.939	407,201	18,064	Unknown	-
10	6.465	71,298	3769	Unknown	-
11	7.478	524,542	19,375	ECG	2.50 ± 0.01

Abbreviation: C = catechin, CF = caffeine, EC = epicatechin, ECG = epicatechin 3-gallate, EGCG = epigallocatechin 3-gallate, GA = gallic acid, GCG = gallocatechin gallate, ND = not detectable, PA = peak area, PH = peak height, T_R_ = retention time. Unknown represents compounds that were detected but not identified.

## Data Availability

The original data presented in this study are included in the article/Supplementary Materials. Further inquiries can be directed to the corresponding author.

## Supplementary Materials

The following supporting information can be downloaded at: https://www.mdpi.com/article/10.3390/antiox14121483/s1. Figure S1. Mass spectra of different phenolic substances obtained by the HPLC-DAD–MS.

## Abbreviations

The following abbreviations/symbols are used in this manuscript:

AA	L-ascorbic acid
ABTS	2,2-Azino-*bis*-(3-methylbenzothiazoline-6-sulfonic acid
ABTS^•+^	ABTS radical
ACSL4	Acyl CoA synthetase long chain family member 4
AIPH	2,2-Azobis-[2-(2-imidazolin-2-yl)propane]dihydrochloride
ANOVA	Analysis of variance
BSA	Bovine serum albumin
C	Catechin
CF	Caffeine
CV	Coefficient of variation
CoQ10	Coenzyme Q10
DAD	Diode array detector
DCFH-DA	2′,7′-Dichlorohydrofluorescein diacetate
DFP	Deferiprone
DI	Deionized water
DMEM	Dulbecco’s modified Eagle medium
[DMEM + FAC]	Iron-loaded DMEM
DMSO	Dimethyl sulfoxide
DTNB	5,5-Dithio-bis (2-nitrobenzoic acid)
EC	Epicatechin
ECG	Epicatechin 3-gallate
EGC	Epigallocatechin
EGCG	Epigallocatechin-3-gallate
ERA	Erastin
FAC	Ferrous ammonium citrate
FAS	Ferrous ammonium sulfate
FBS	Fetal bovine serum
FO	FerroOrange
Fe^2+^	Ferrous ion
Fer-1	Ferrostatin 1
FI	Fluorescence intensity
*FTH**/**L*	Ferritin heavy-chain and light-chain gene
GA	Gallic acid
GAE	Gallic acid equivalent
GCG	Gallocatechin gallate
GPX-4	Glutathione peroxidase 4
GR	Glutathione reductase
GSH	Glutathione
GSSG	Oxidized glutathione
GTE	Green tea extract
H_2_DCFH-DA	2′,7′-Dichlorohydrofluorescein diacetate reduced form
HEPES	Hydroxyethyl piperazine ethane sulfonic acid
*HFE*	*High Fe^2+^* gene
4-HNE	4-Hydroxynonenal
Huh7	Human hepatocellular carcinoma
IC_50_	Half-maximal inhibitory concentration
ip	Intraperitoneal
LIP	Labile iron pool
Lipro-1	Liproxstatin-1
LOD	Limit of detection
LPO	Lipid hydroperoxides
MDA	Malondialdehyde
MPA	*m*-phosphoric acid
MTT	3-(4,5-Dimethylthiazol-2-yl)-2,5-diphenyltetrazolium bromide
NAC	*N*-acetylcysteine
NAFLD	Nonalcoholic fatty liver disease
NO_3_	Peroxynitrite
NRF-2	Nuclear factor erythroid 2-related factor 2
OD	Optical density
PB	Phosphate buffer
PBS	Phosphate-buffered saline
PTFE	Polytetrafluoroethylene
QE	Quercetin equivalent
ROS	Reactive oxygen species
RSL-3	RAS-selective lethal 3
SD	Standard deviation
SEM	Standard error of the means
SI	Serum iron
SLC3A2	Solute carrier family 3 member A2
SLC7A11	Solute carrier family 7 member 11
TBA	Thiobarbituric acid
TEAC	Trolox equivalent antioxidant capacity
TFC	Total flavonoid content
TIBC	Total iron–binding capacity
TPC	Total phenolic content
T_R_	Retention time
Trolox	6-Hydroxy-2,5,7,8-tetramethylchroman-2-carboxylic acid
TS	Transferrin saturation
*v*/*v*	Volume by volume
*w*/*v*	Weight by volume
-xCT	Cystine/glutamate transporter
ε	Molar extinction coefficient
λ	Wavelength
^•^	Radical
