# Supplementary figures and images for: Green Tea Catechins Mitigate Hepatocyte Ferroptosis Through Attenuation of Oxidative Stress and Improvement of Antioxidant Systems
Source: Antioxidants (Basel). 2025 Dec 10;14(12):1483. doi: 10.3390/antiox14121483 (PMC12729789; doi:10.3390/antiox14121483)

A

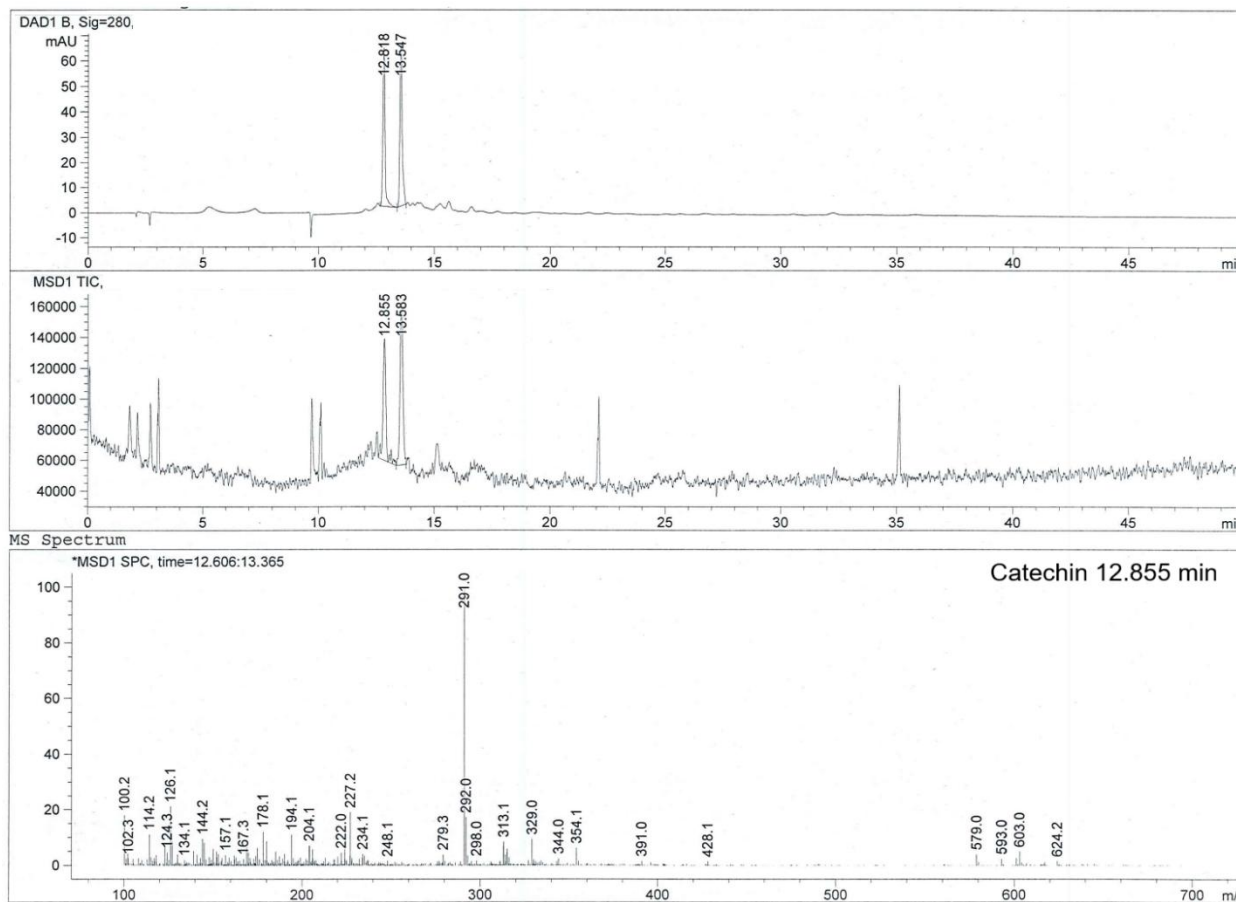

B

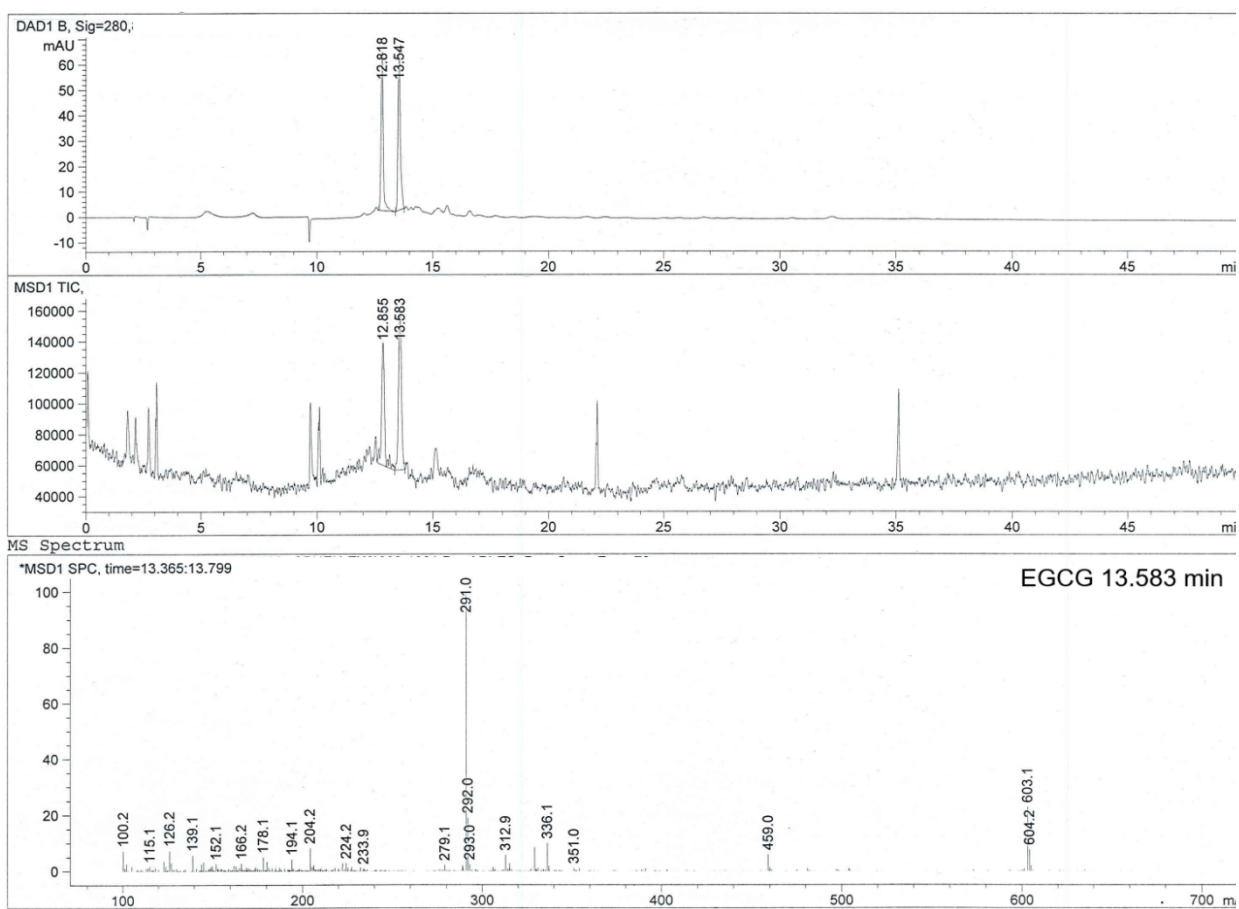

C

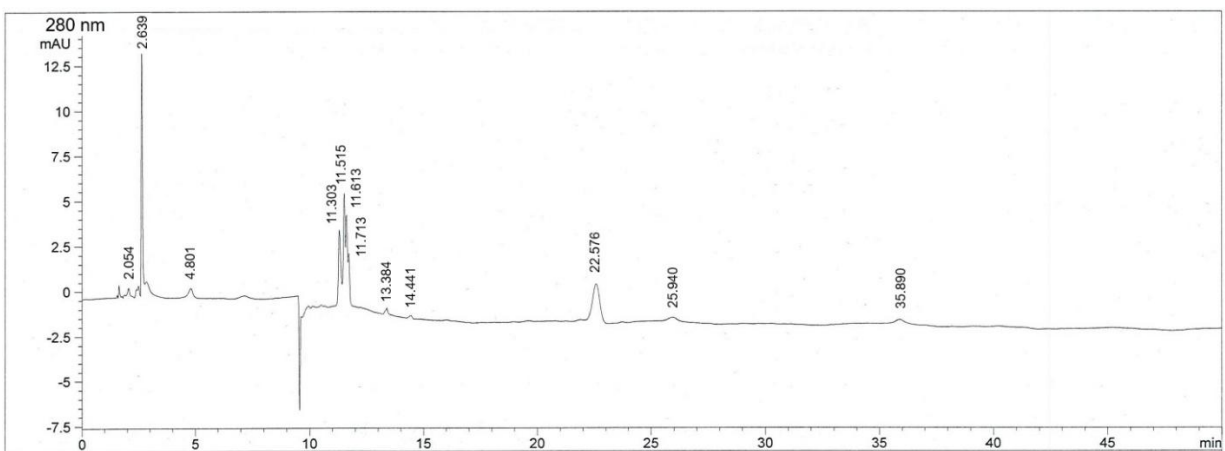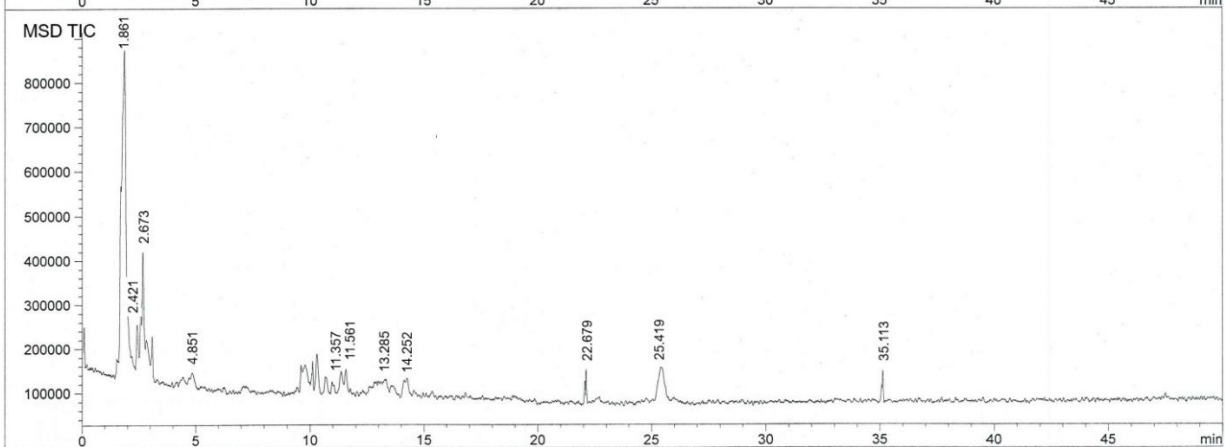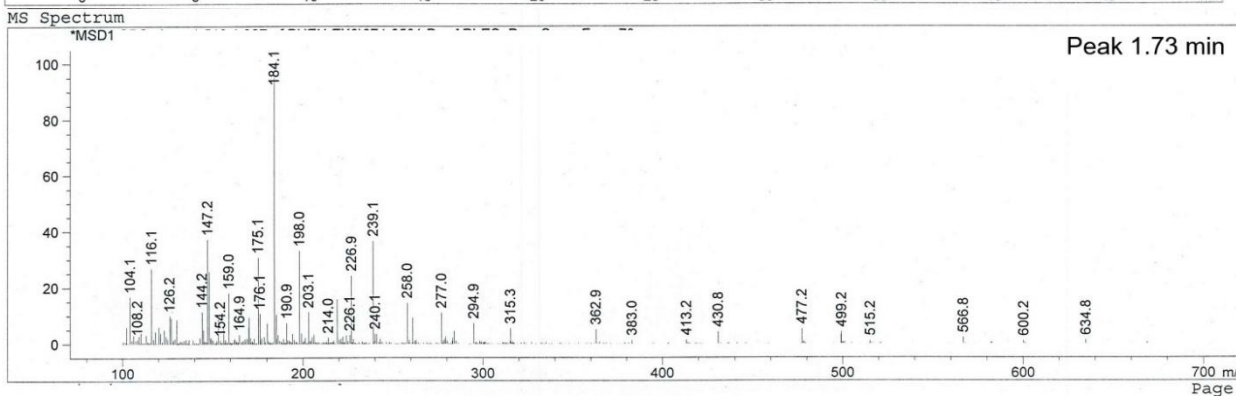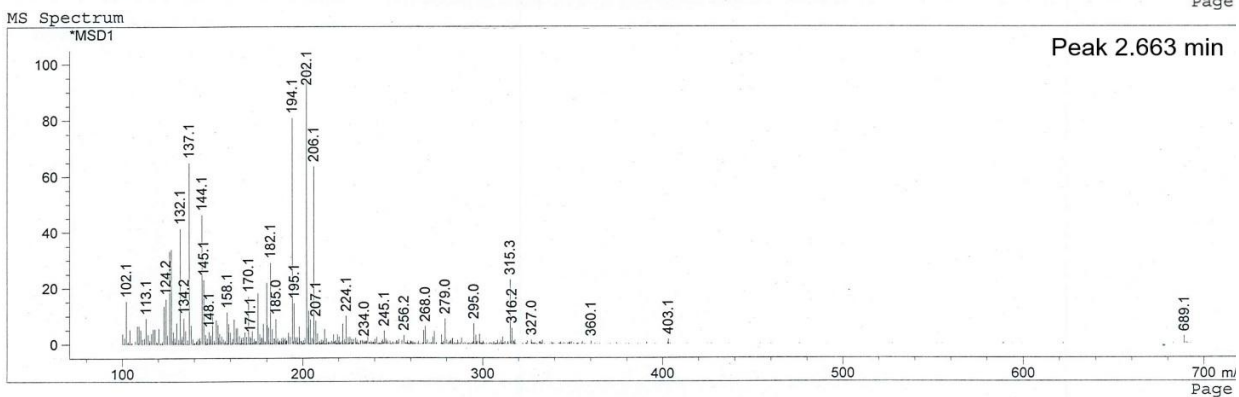

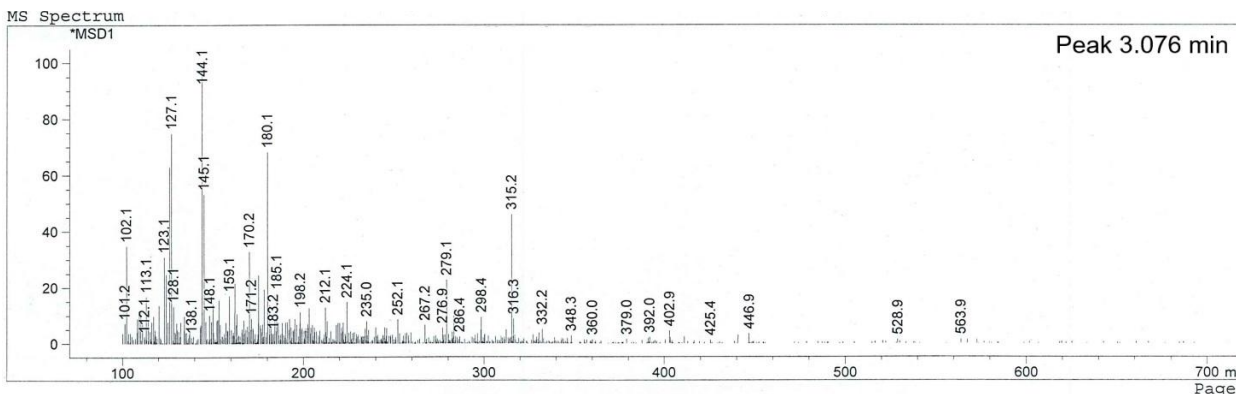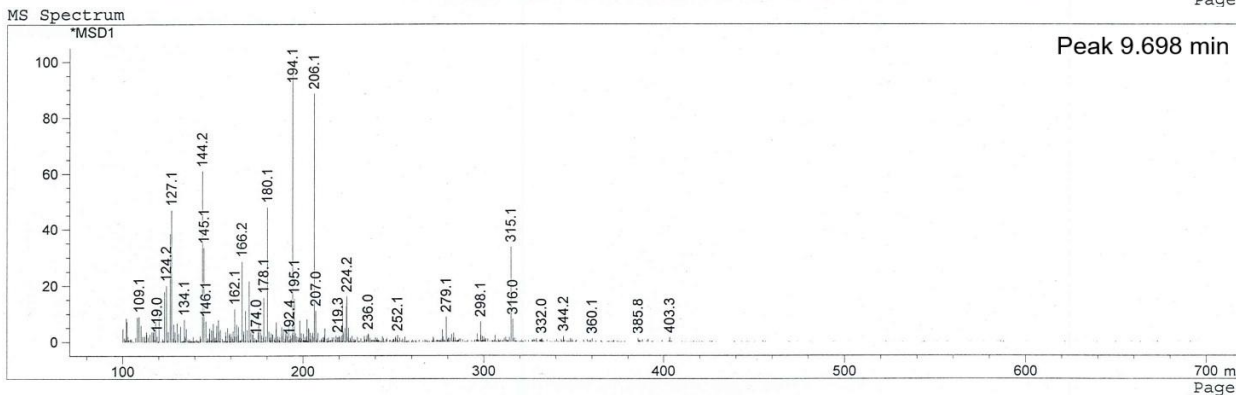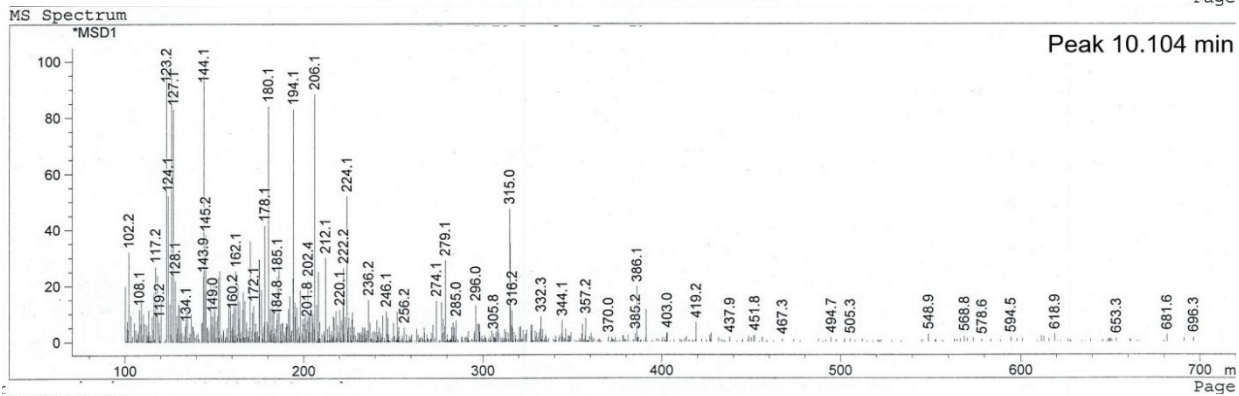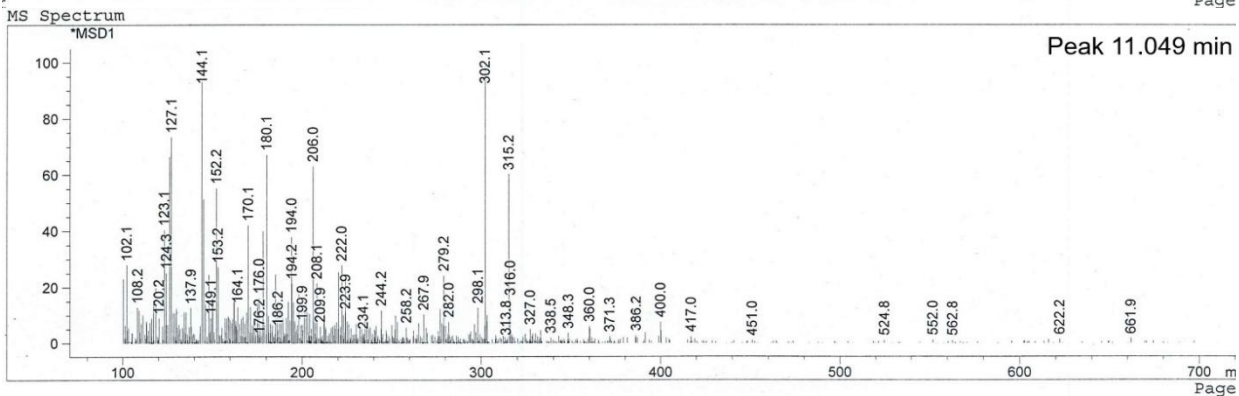

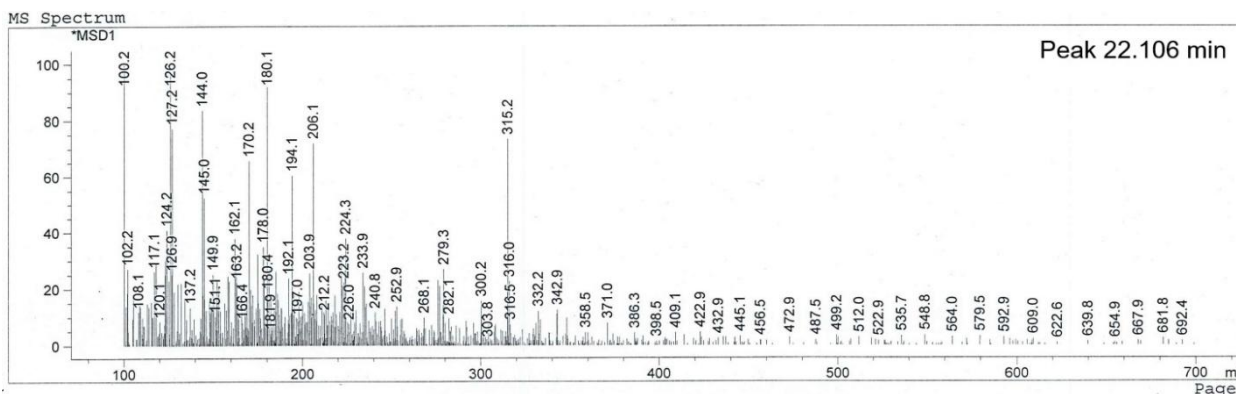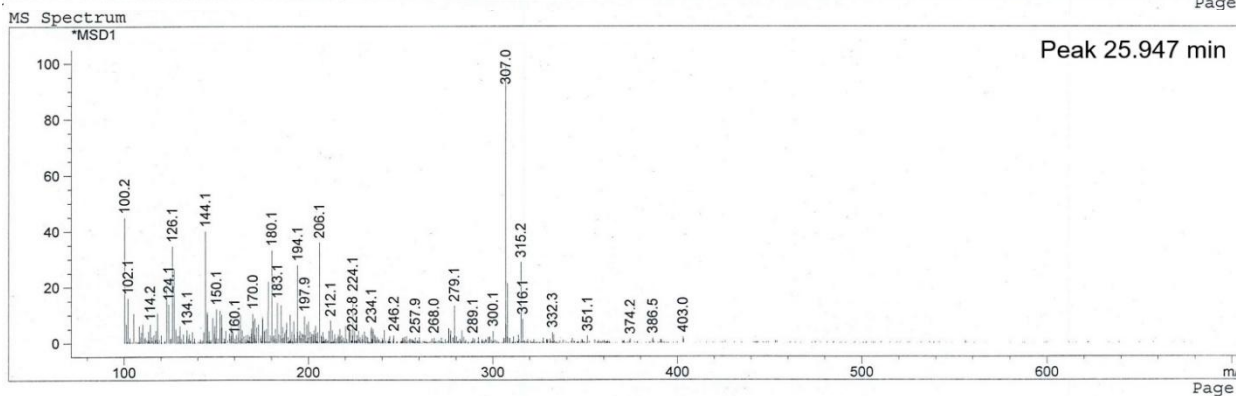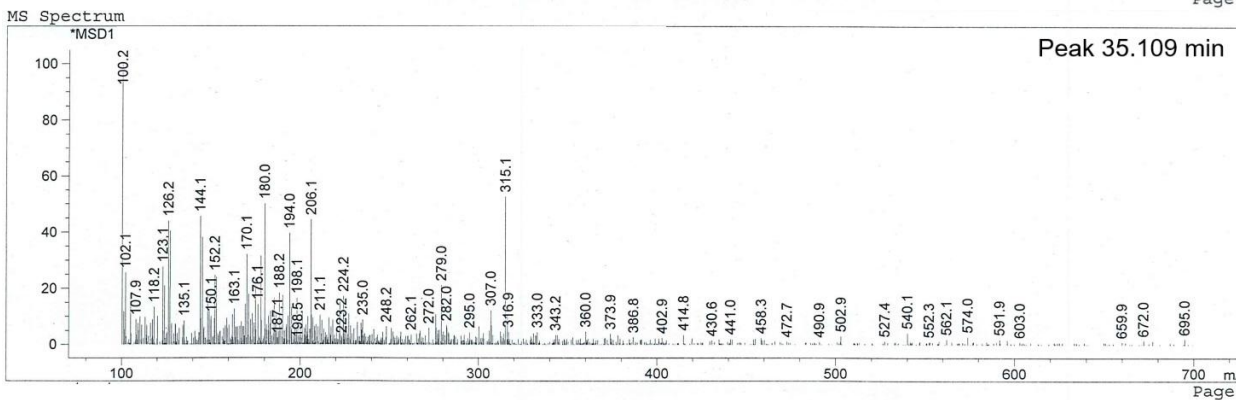

D

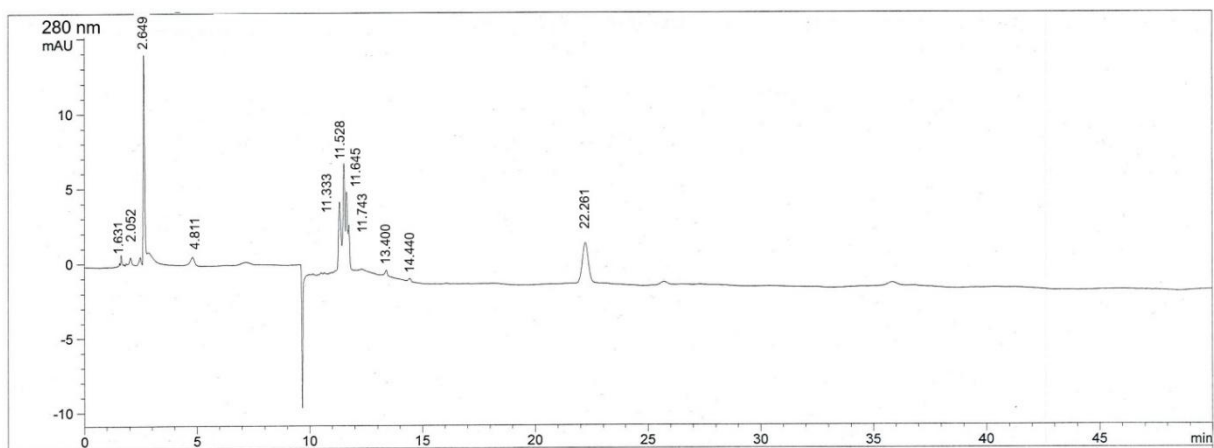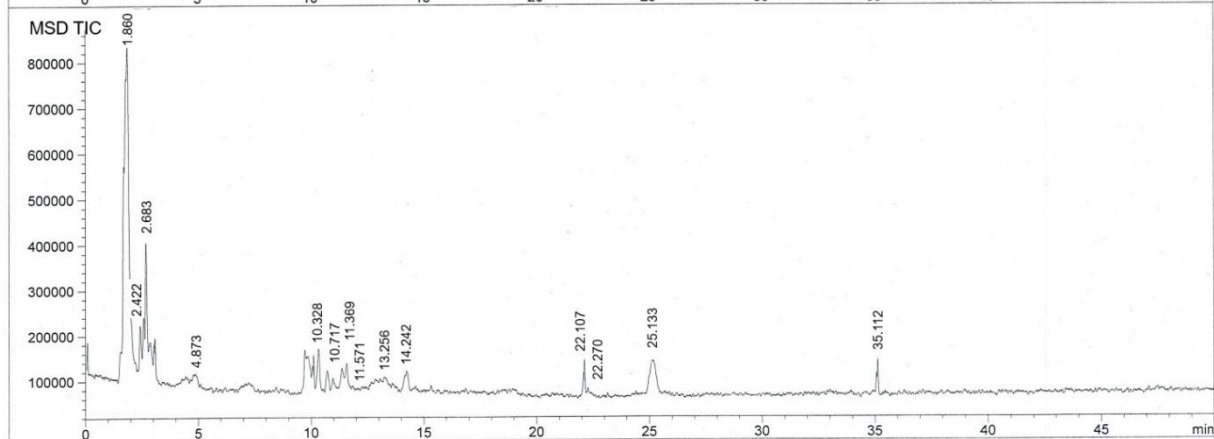

MS Spectrum

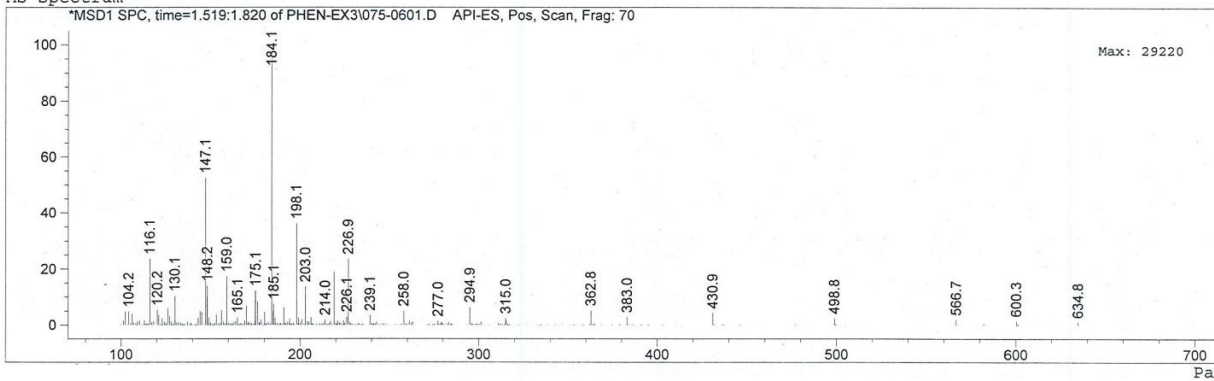

MS Spectrum

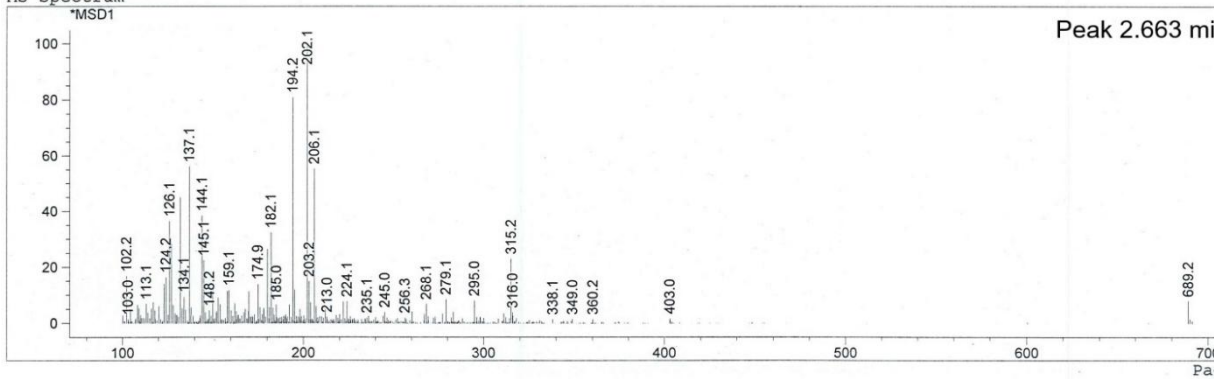

Peak 2.663 min

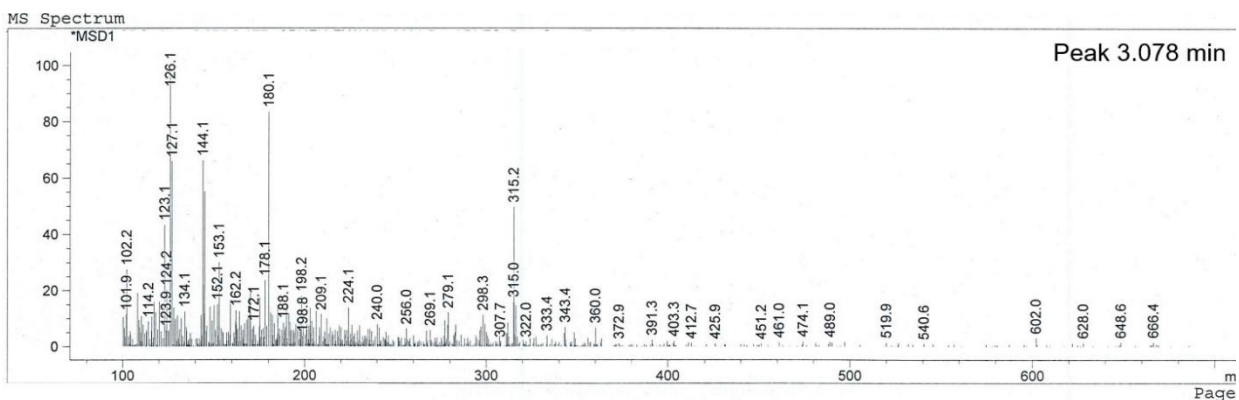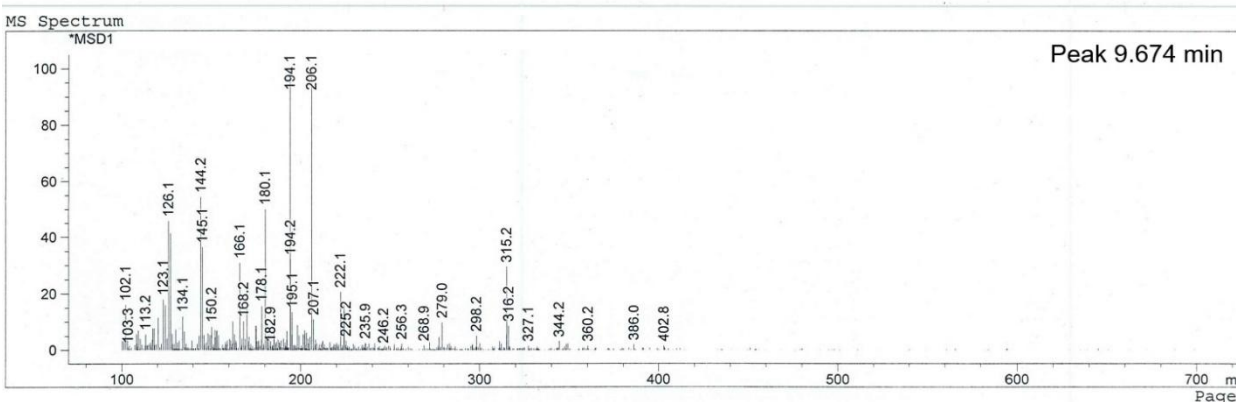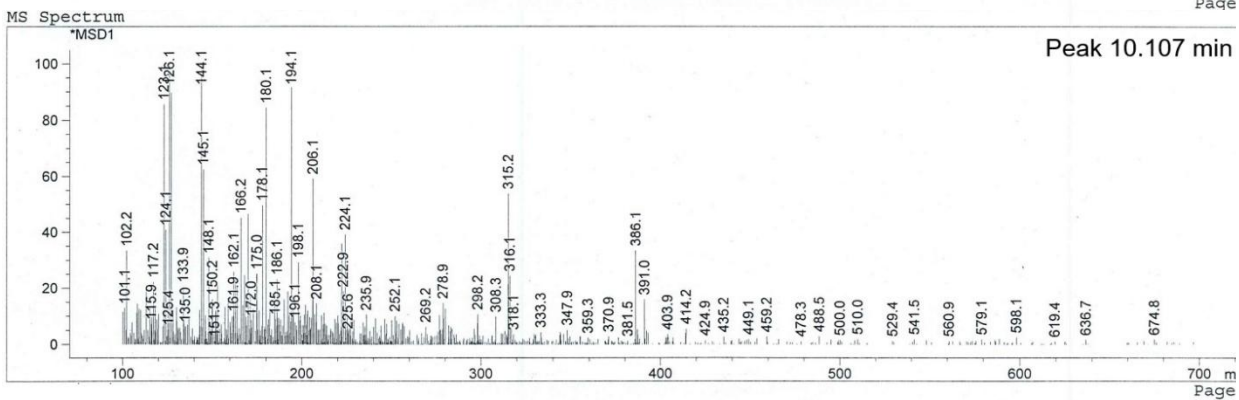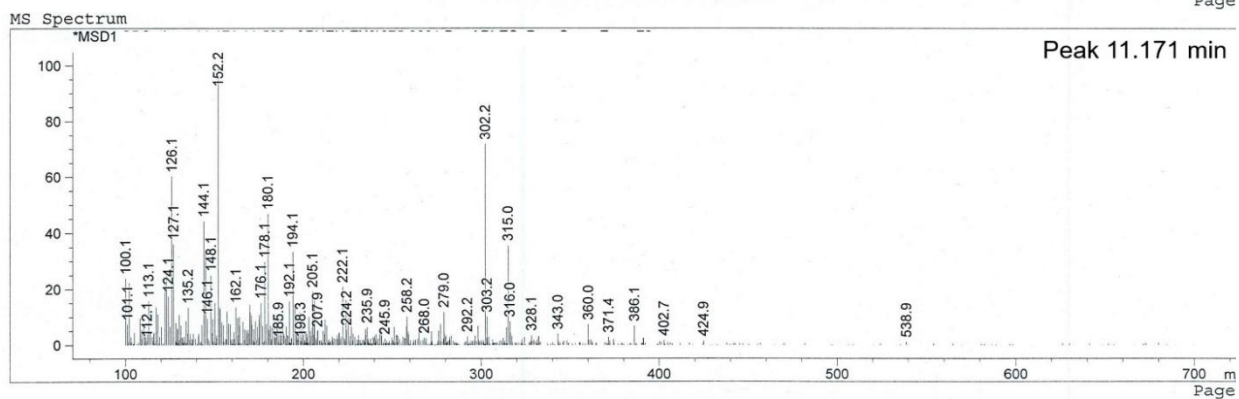

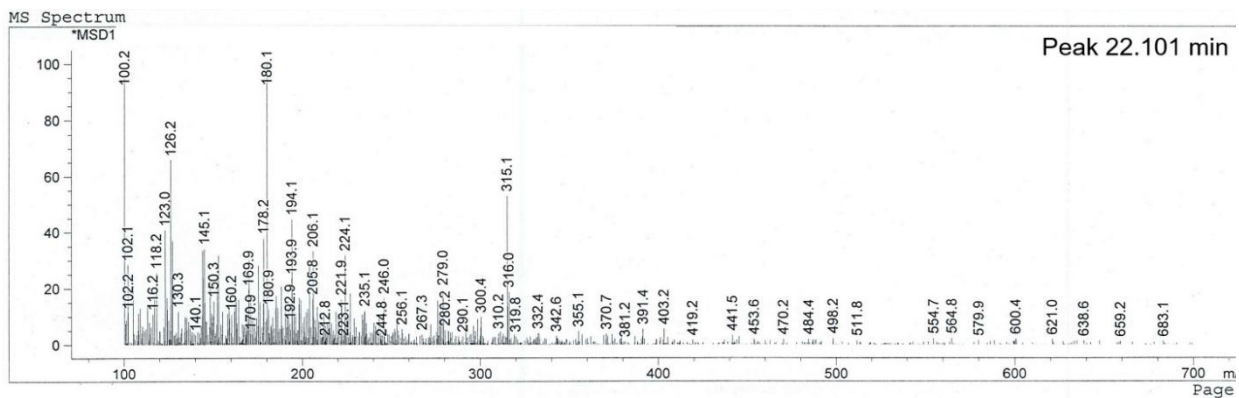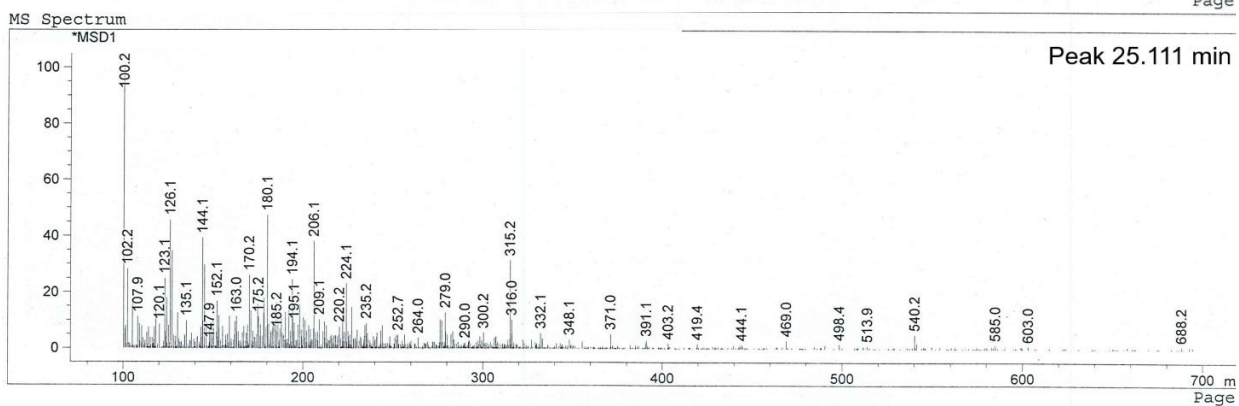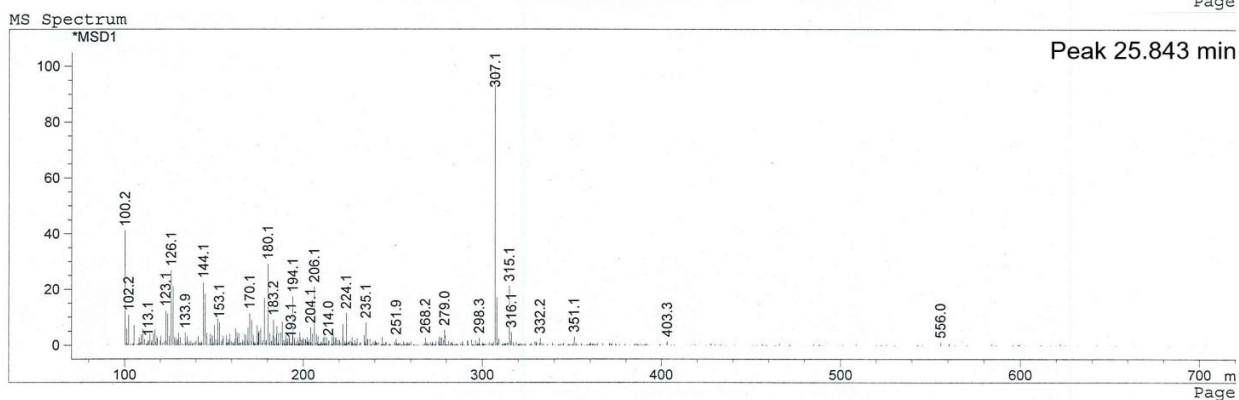

E

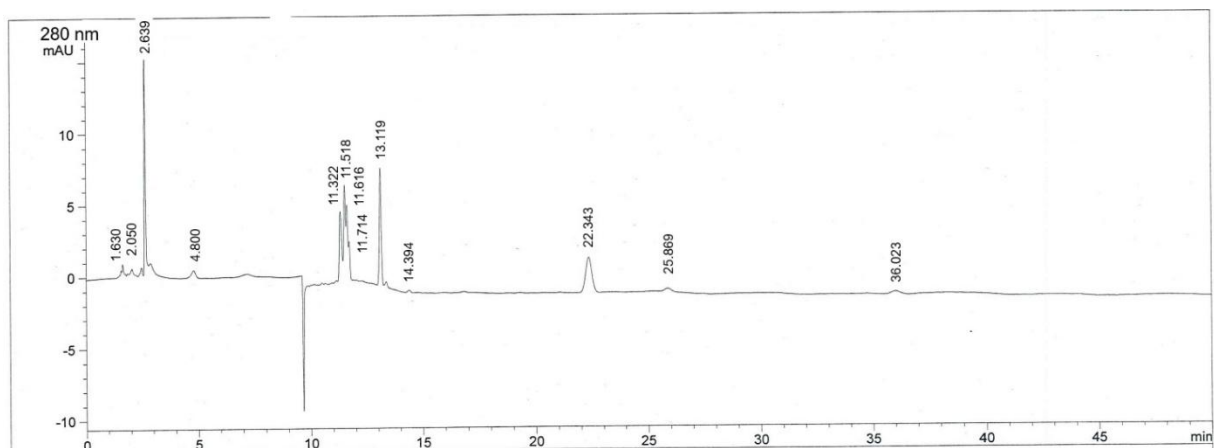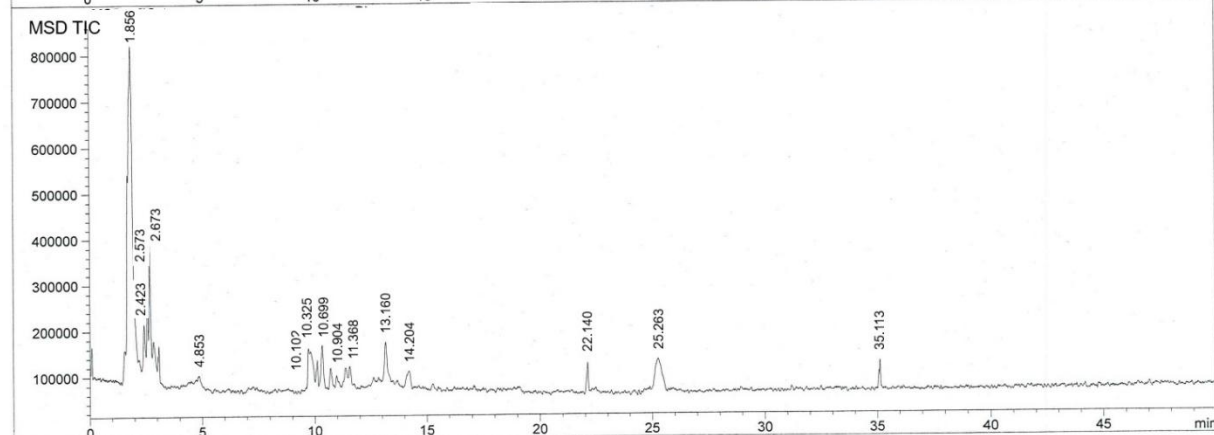

MS Spectrum

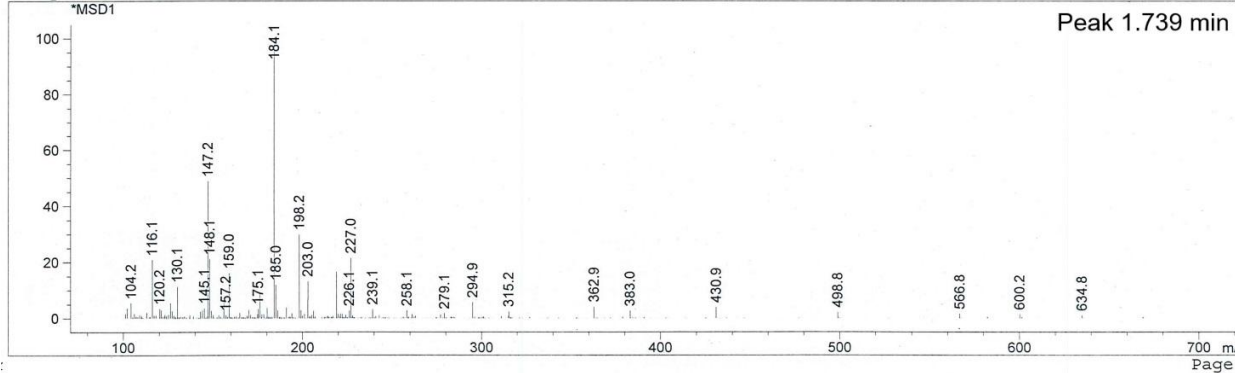

MS Spectrum

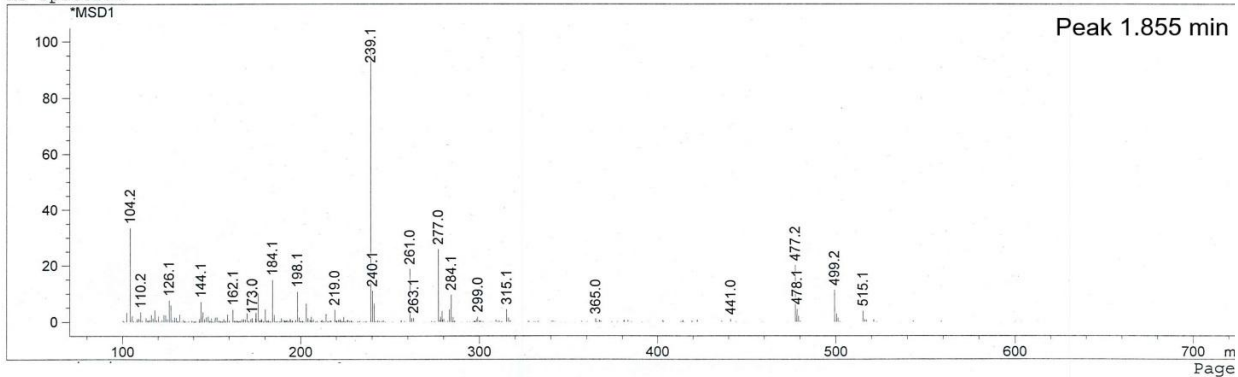

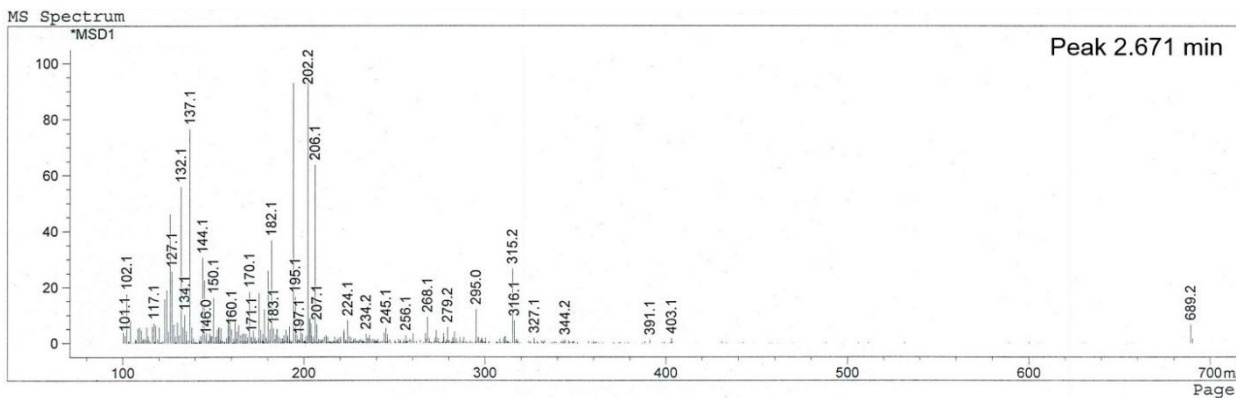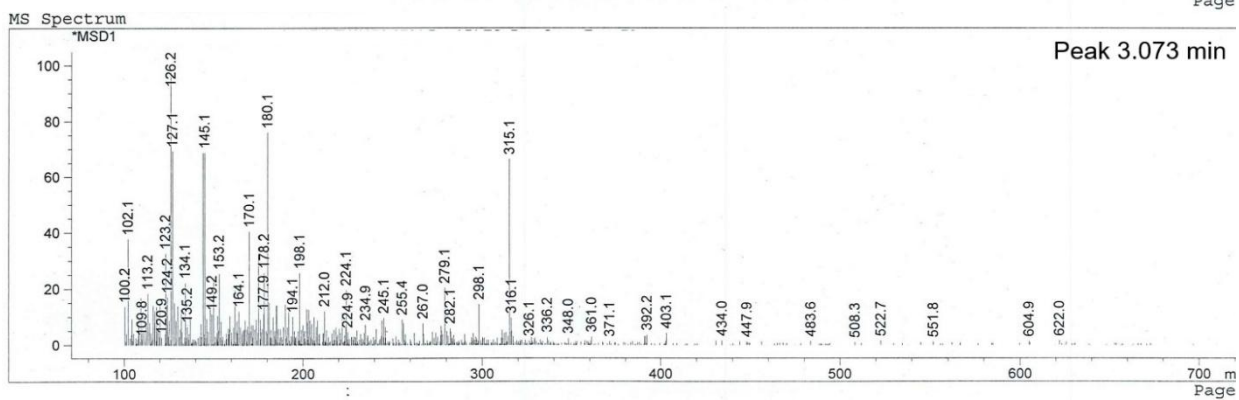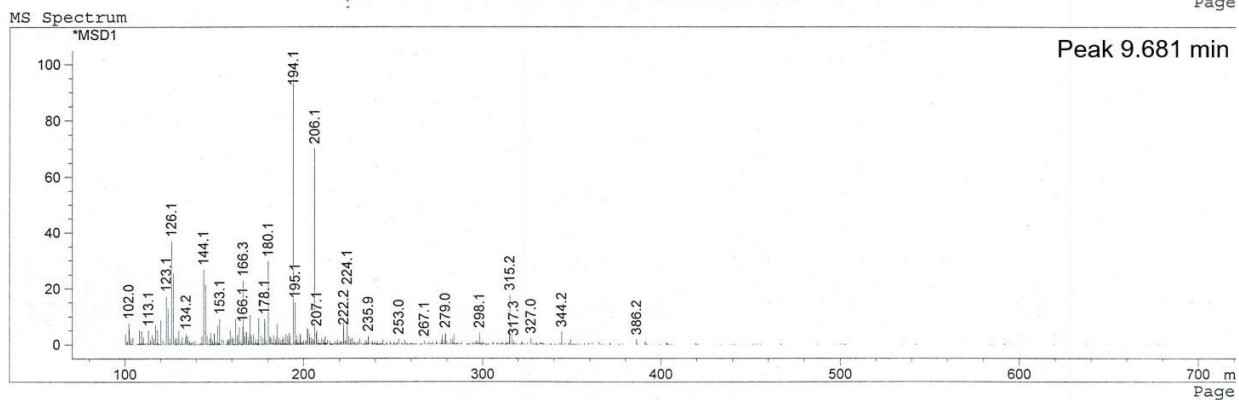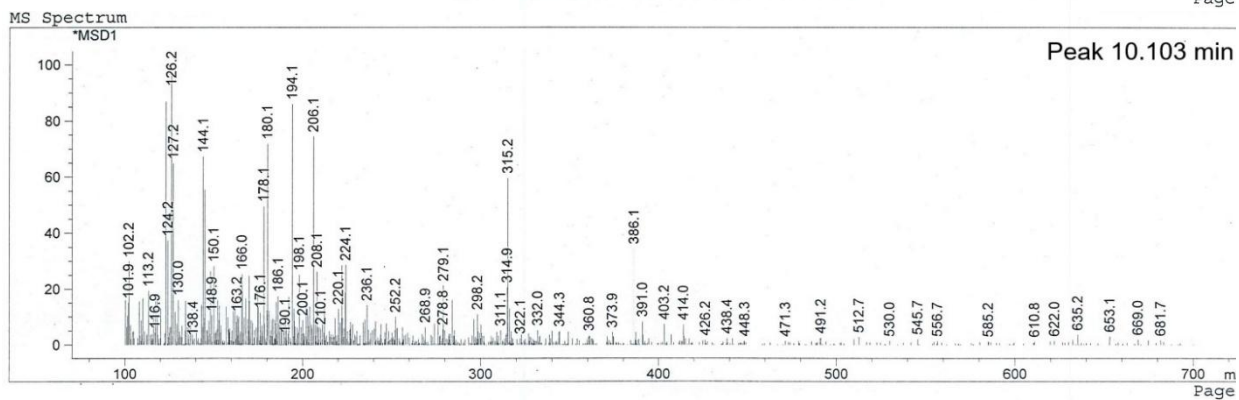

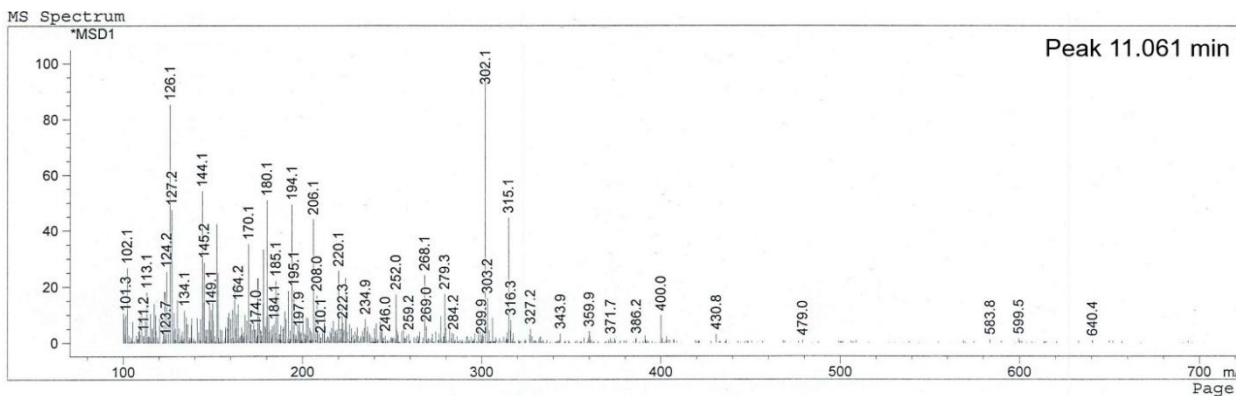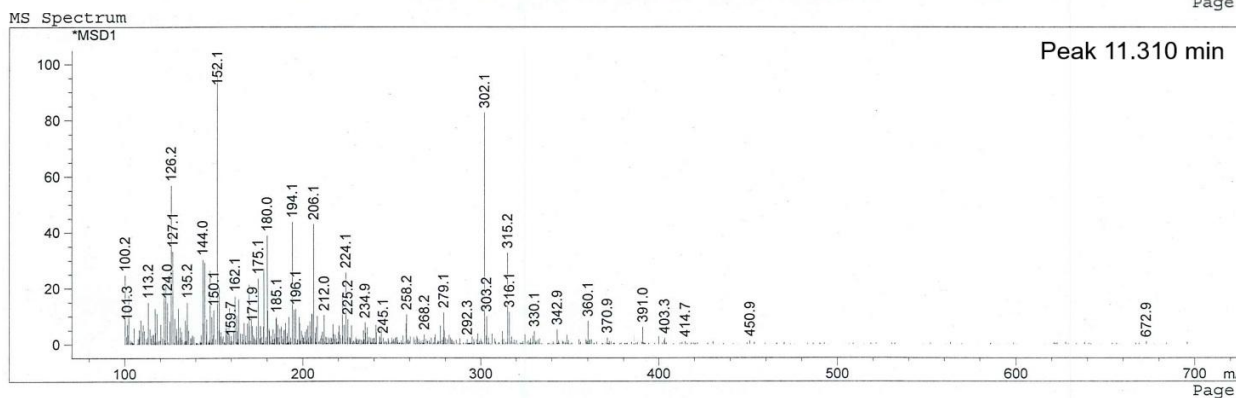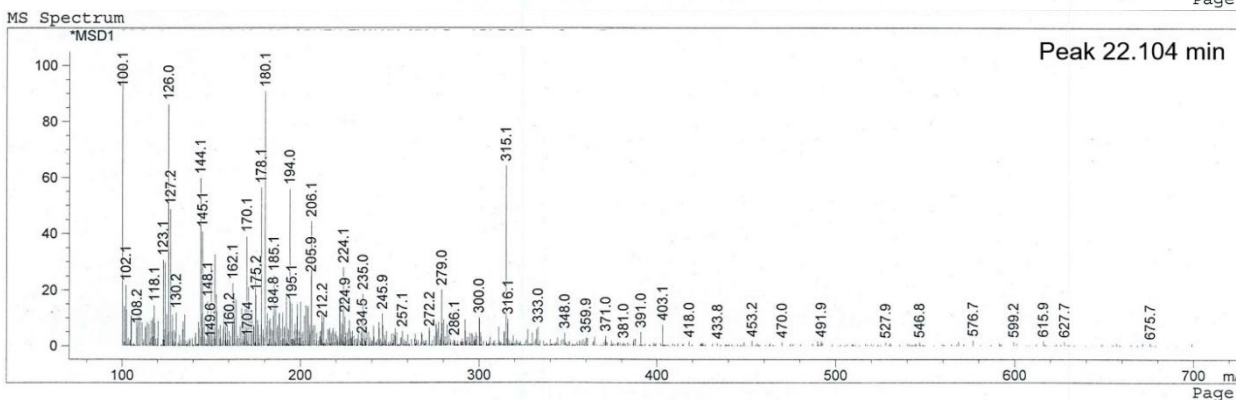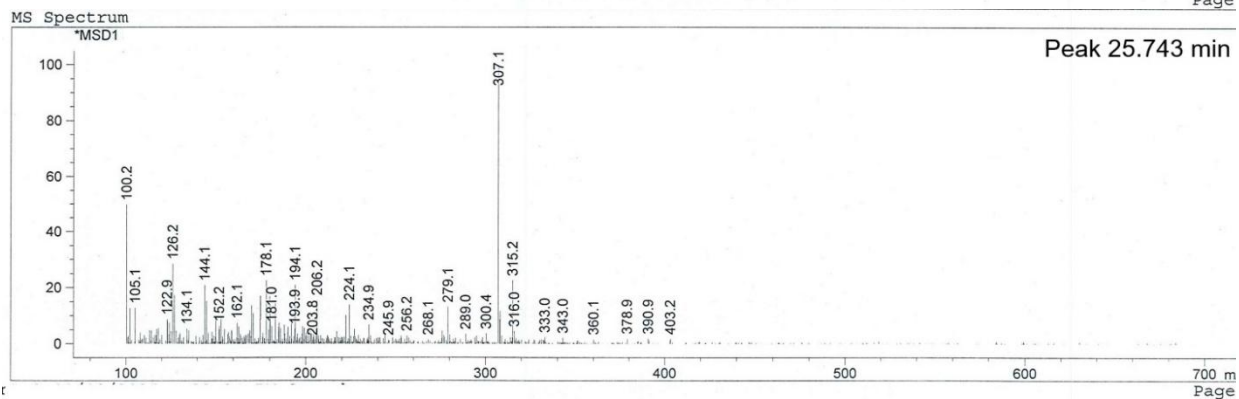

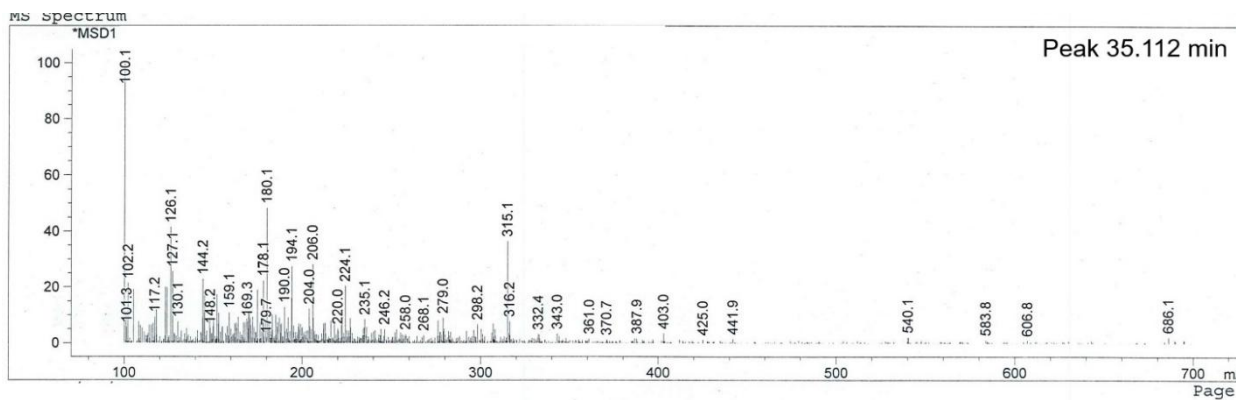

F

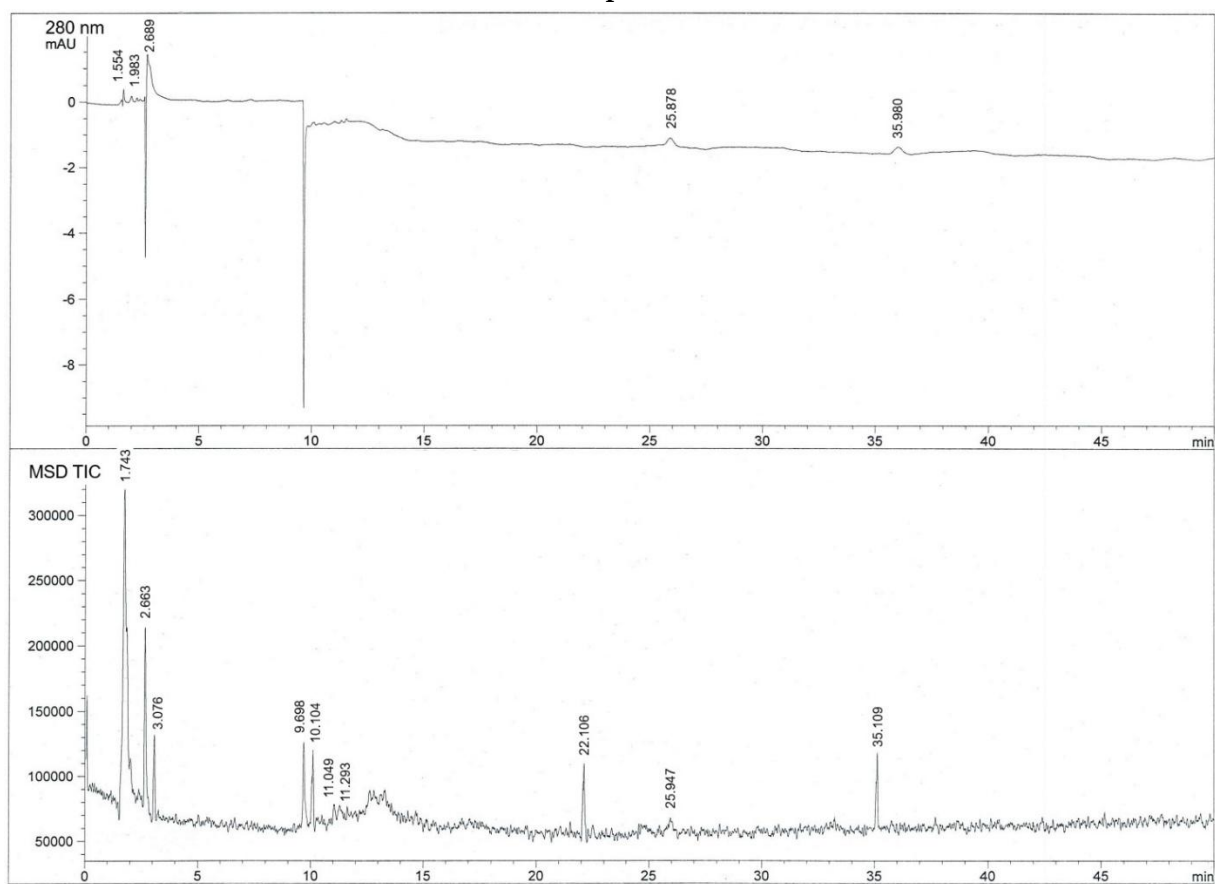

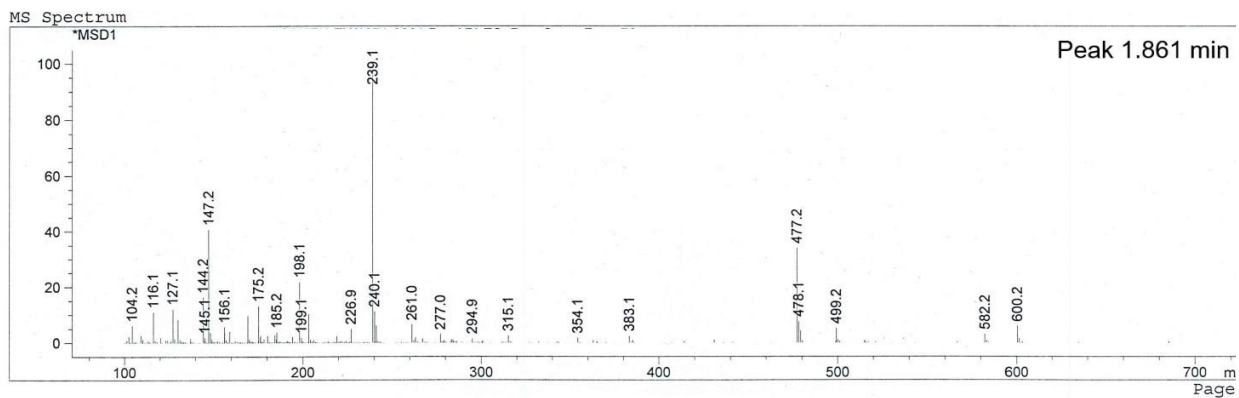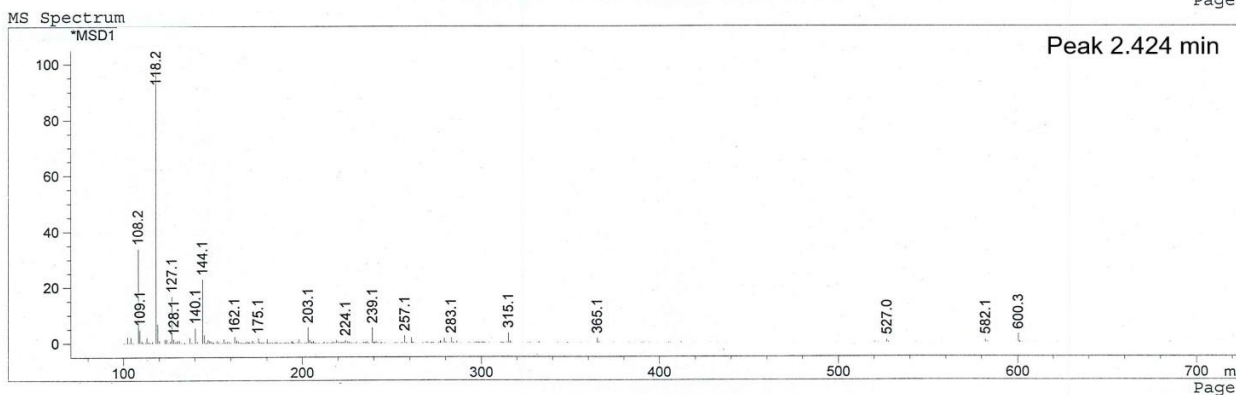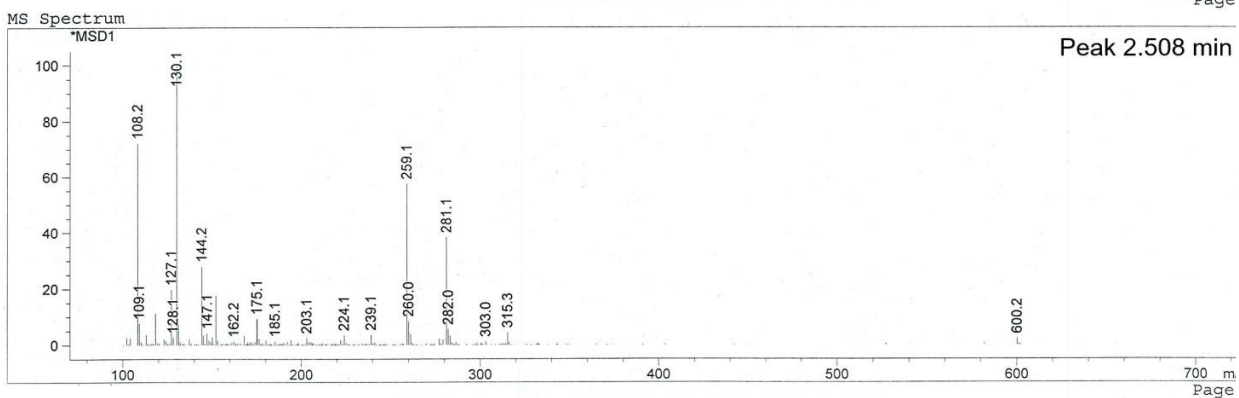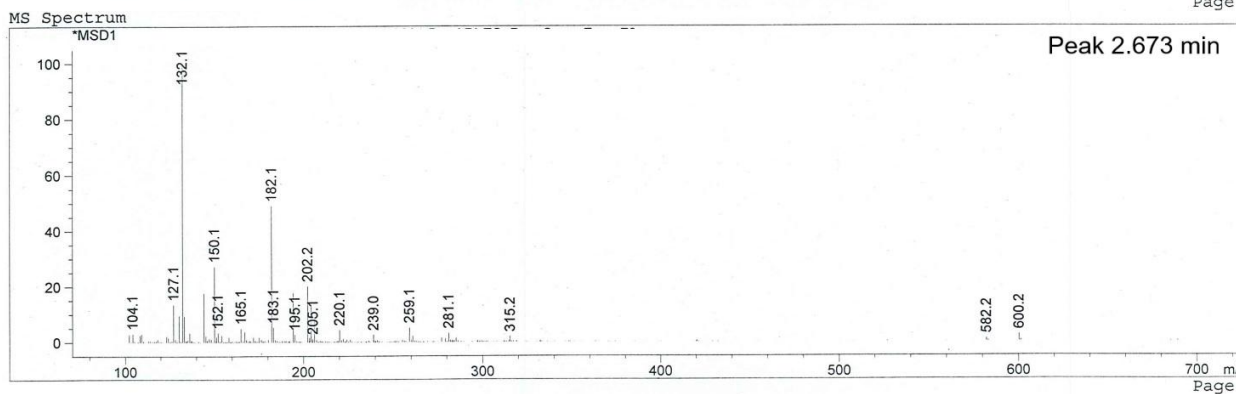

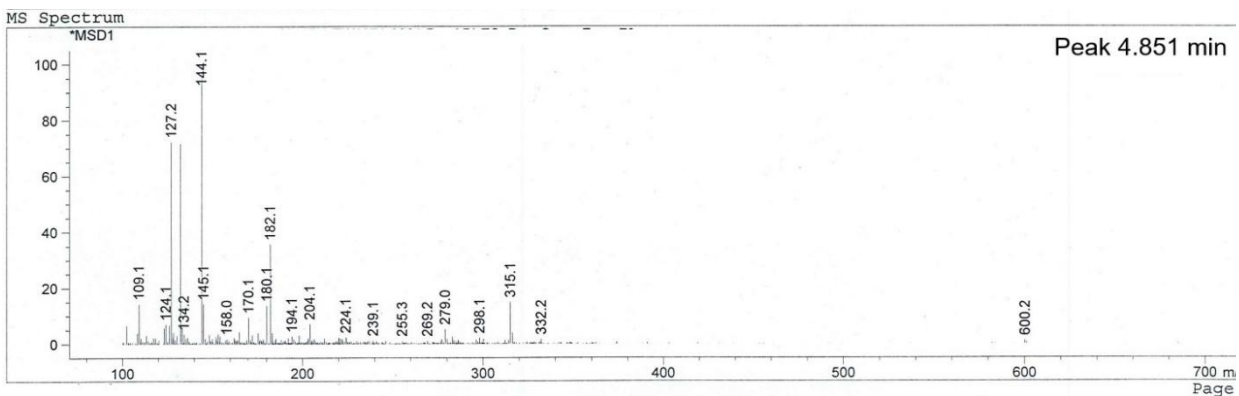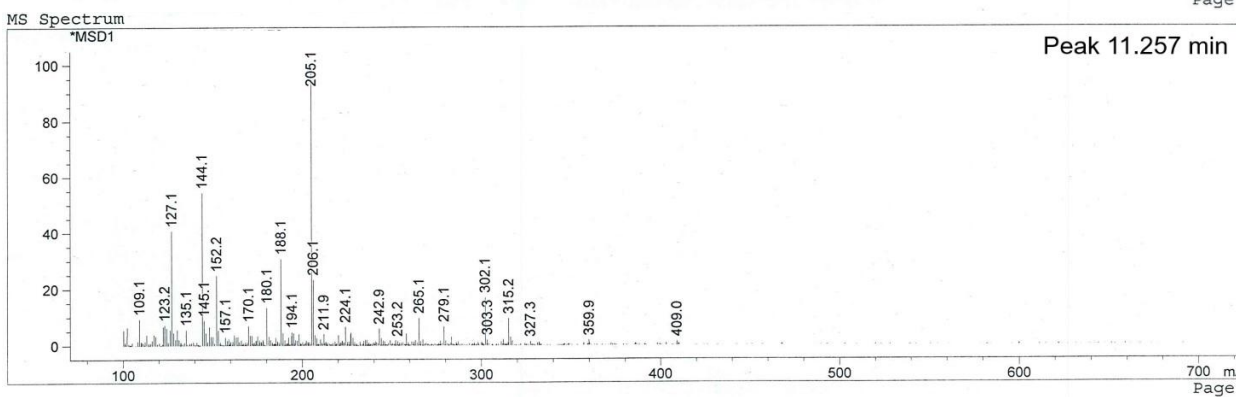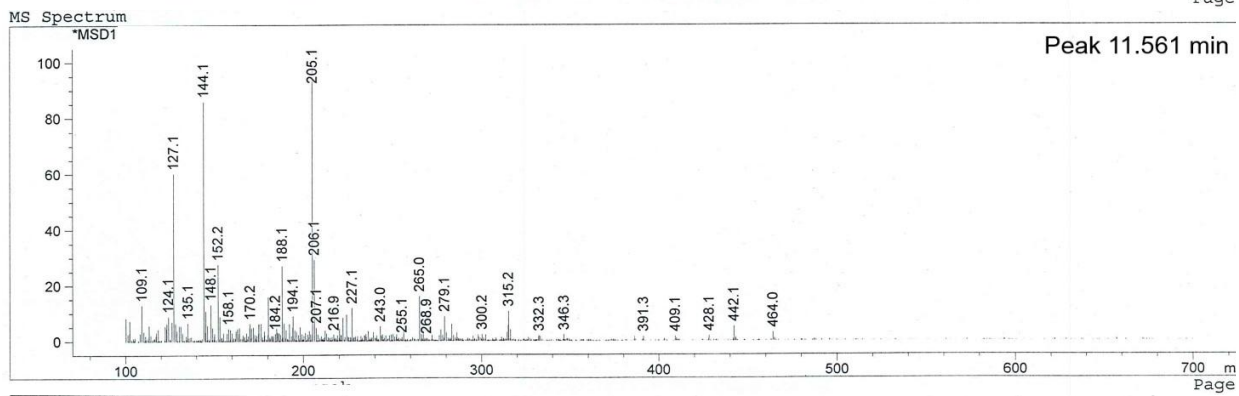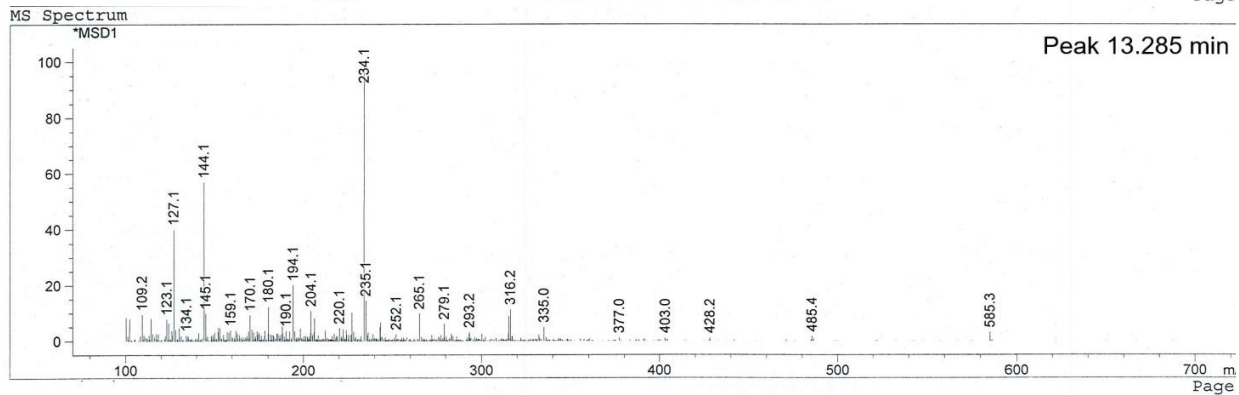

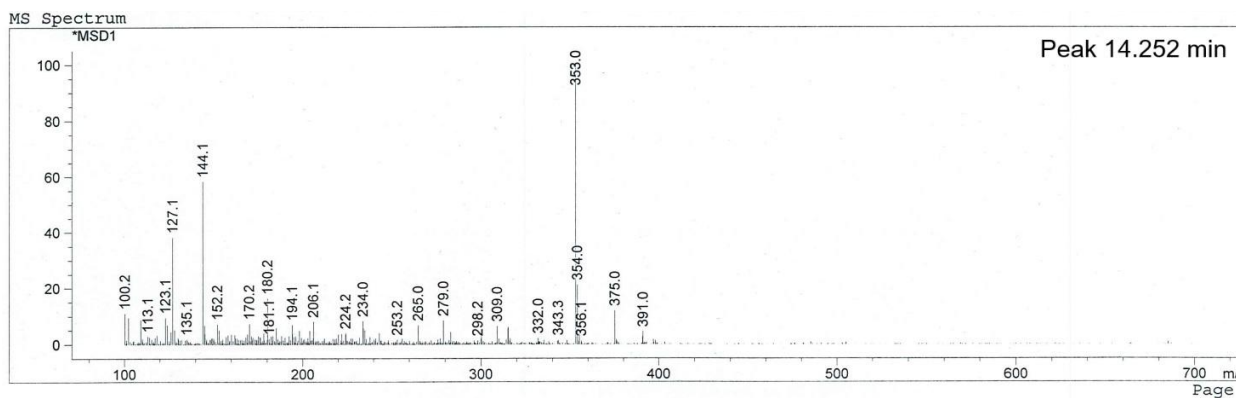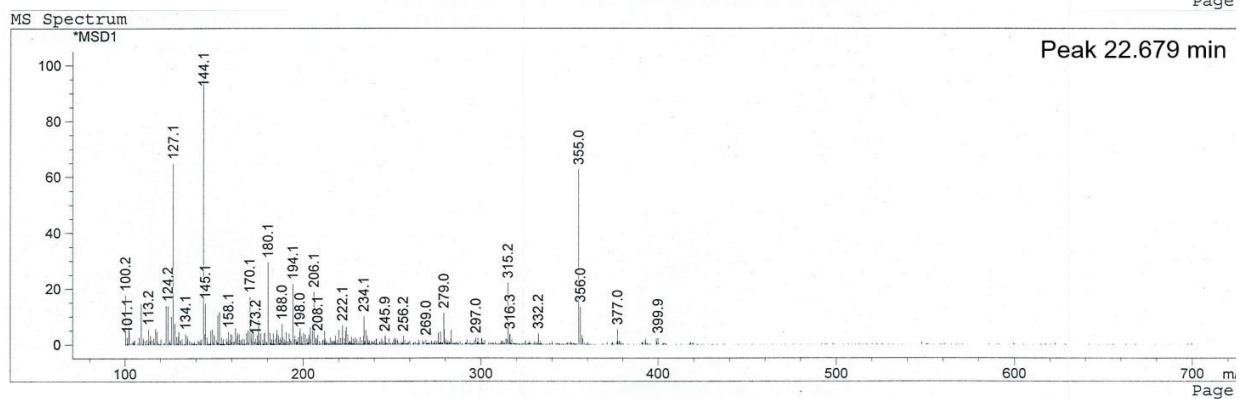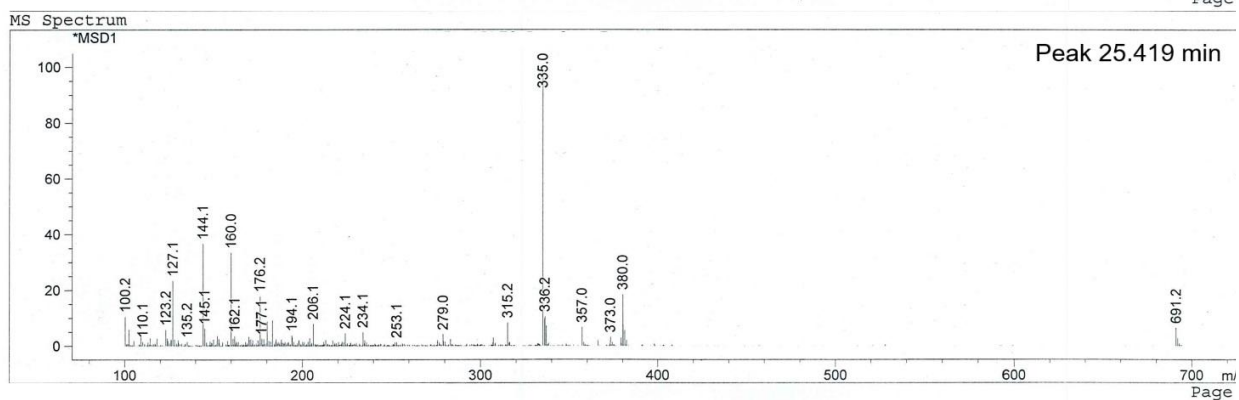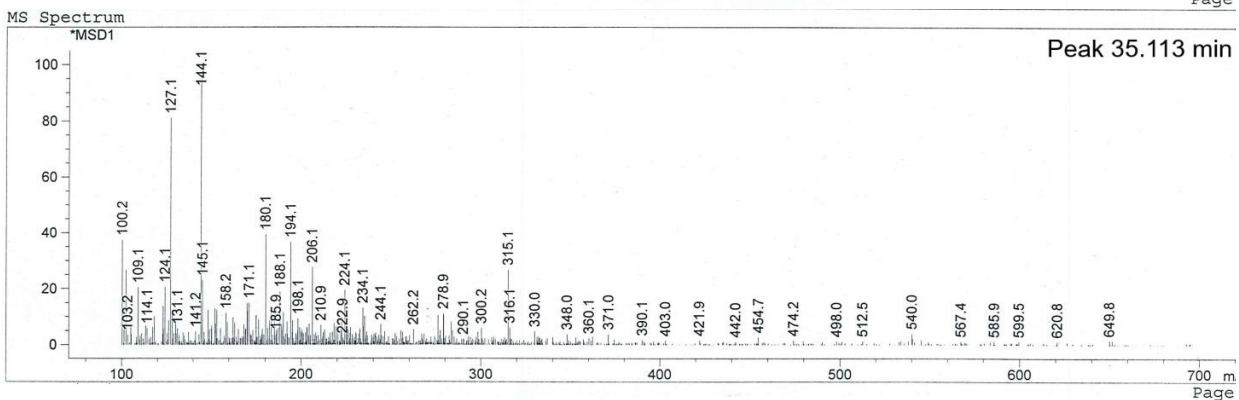

G

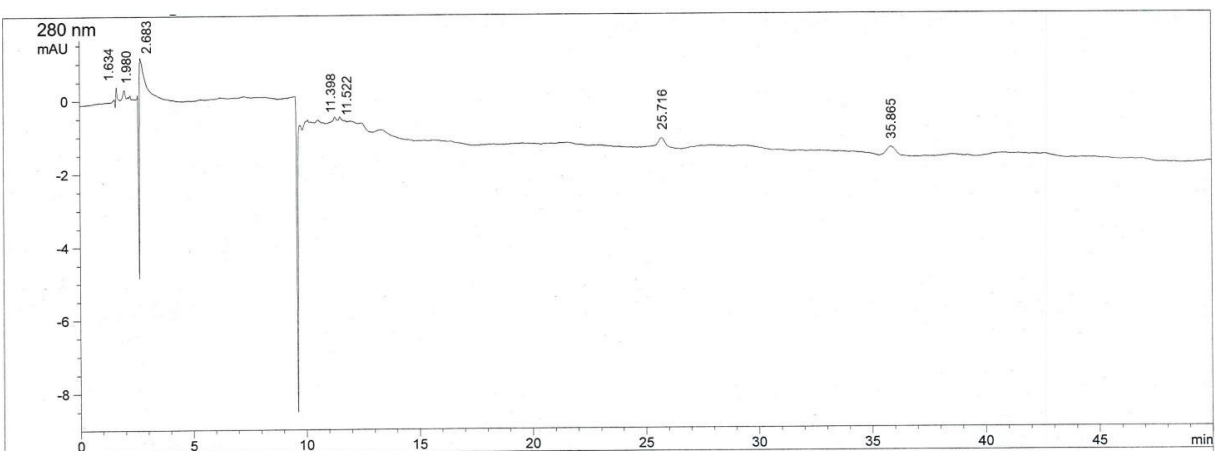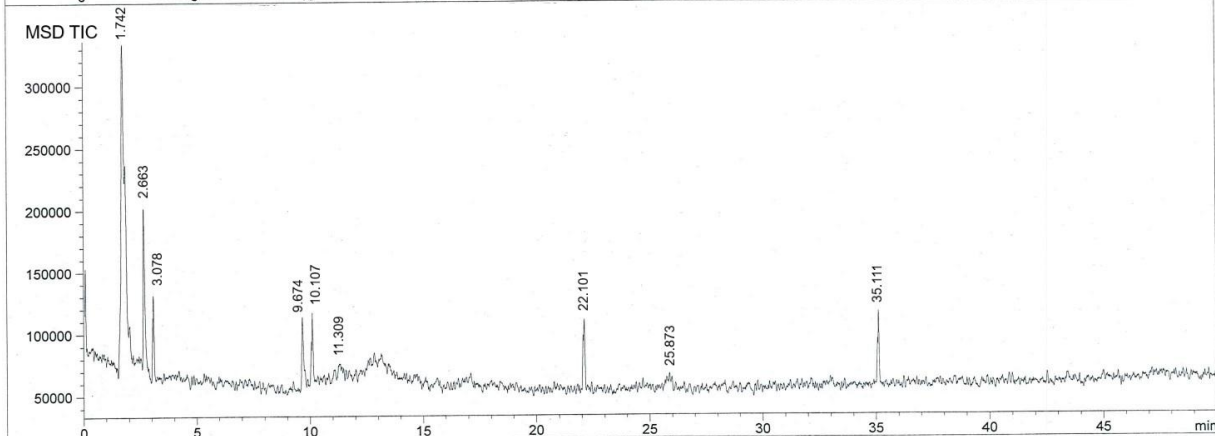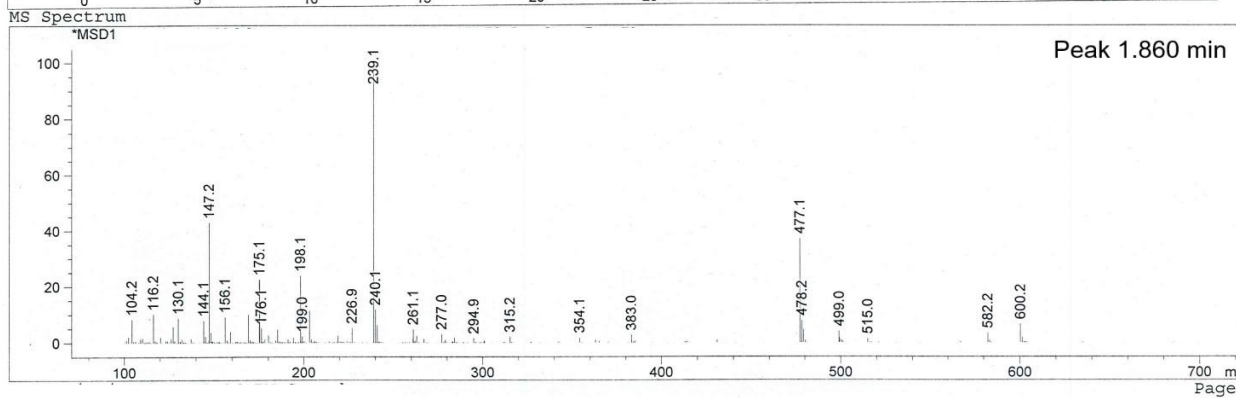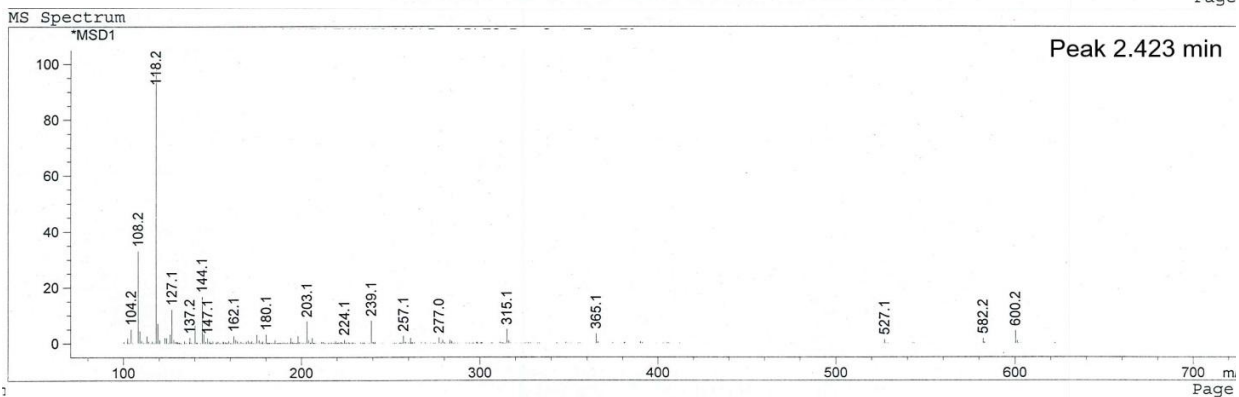

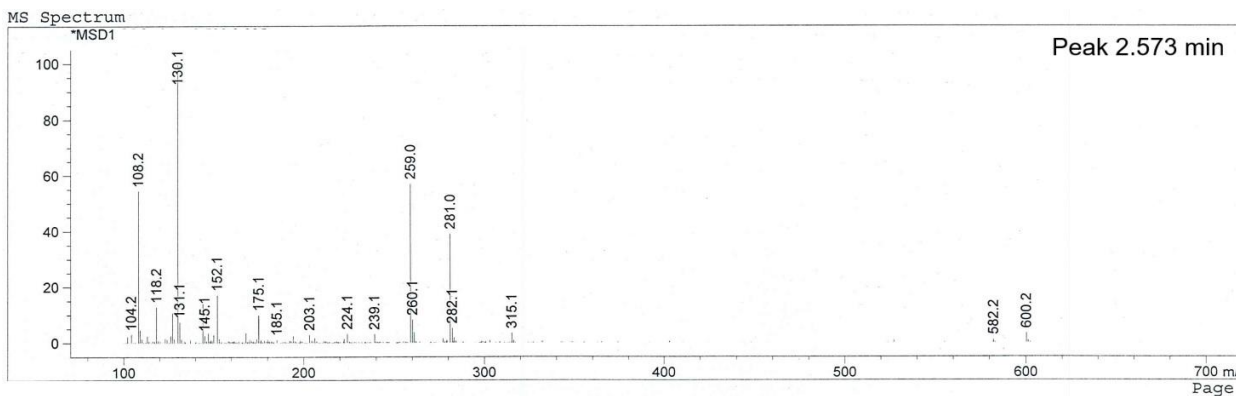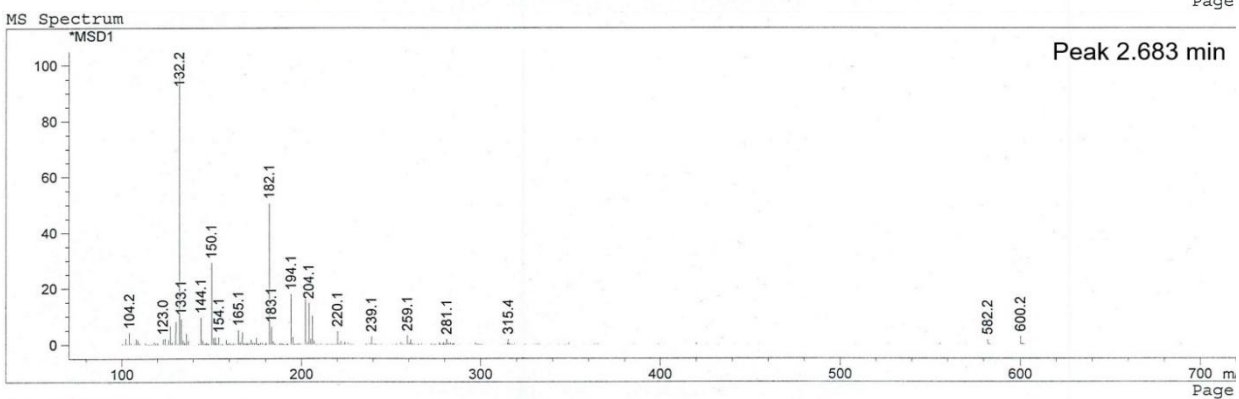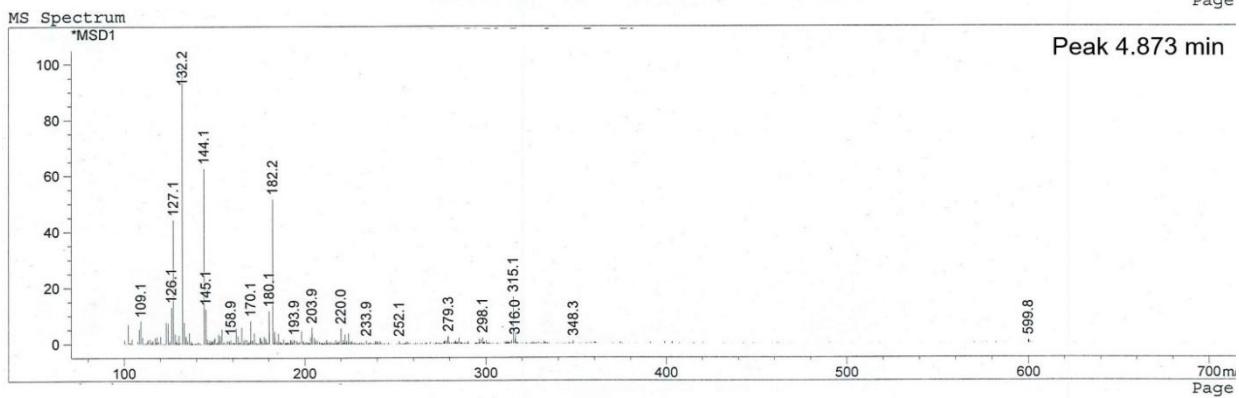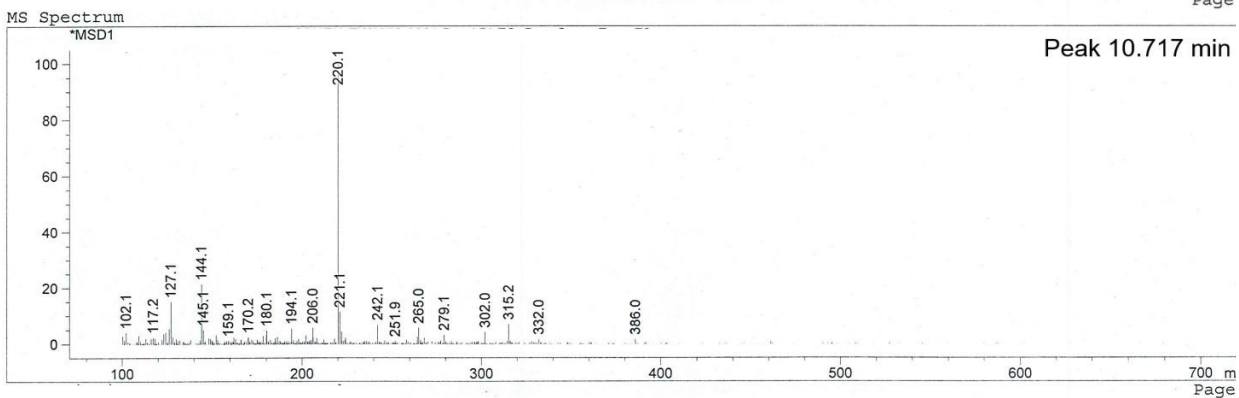

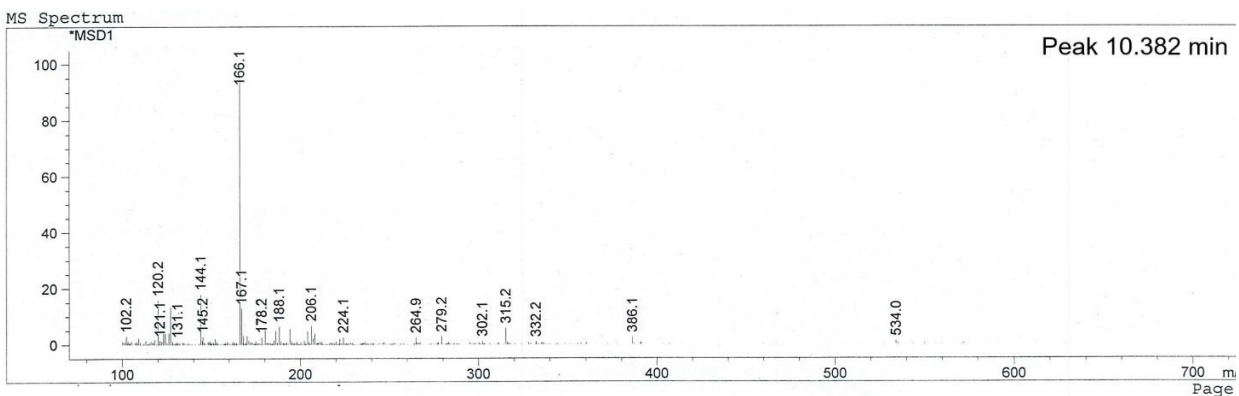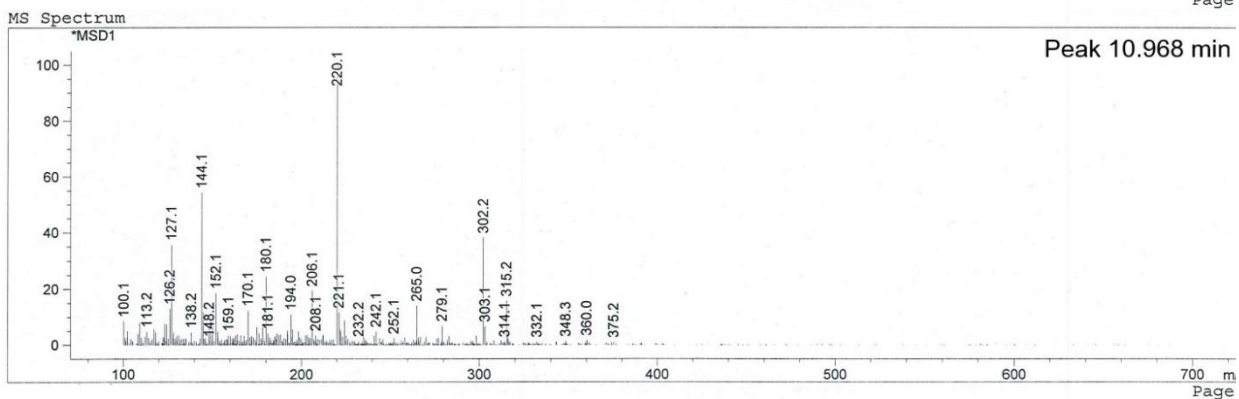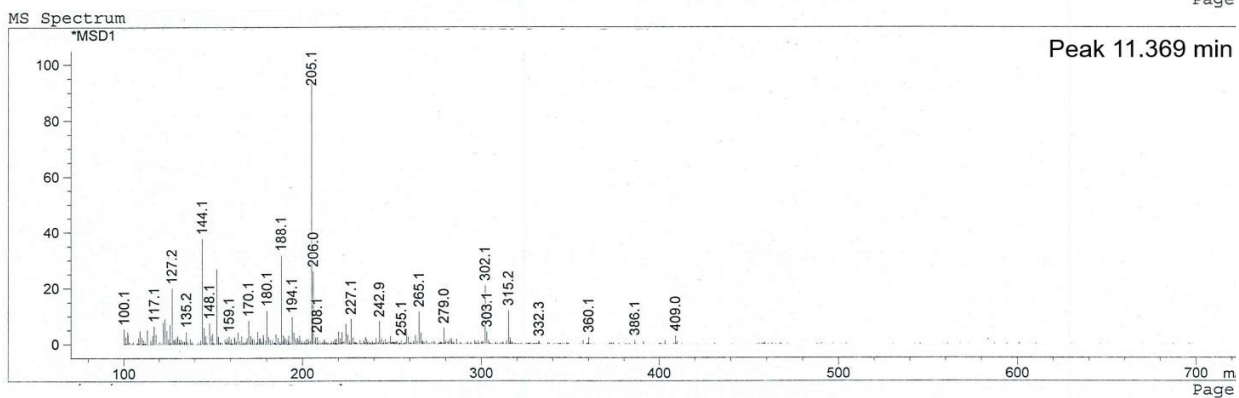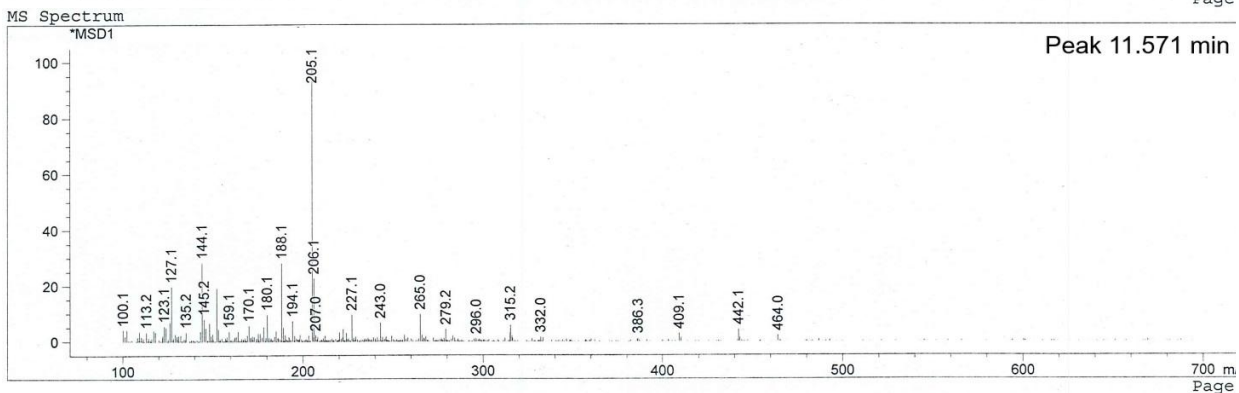

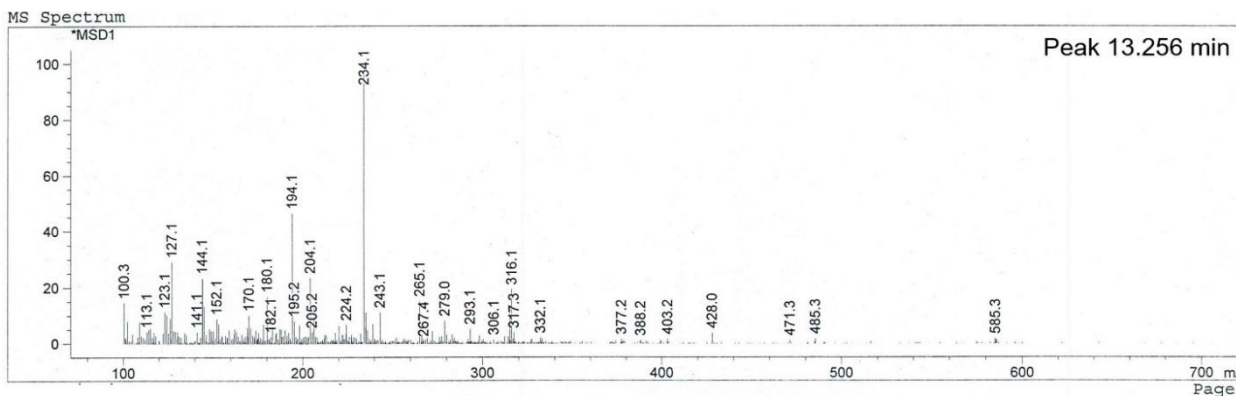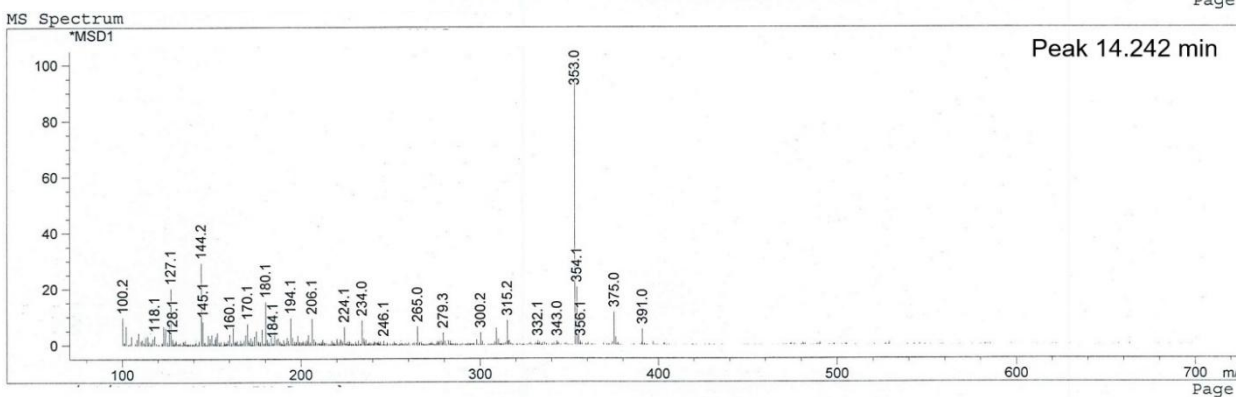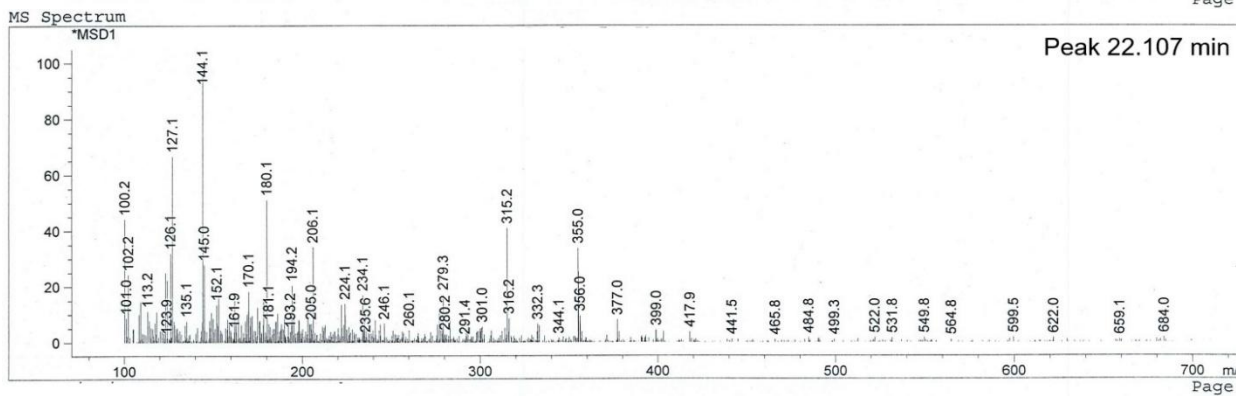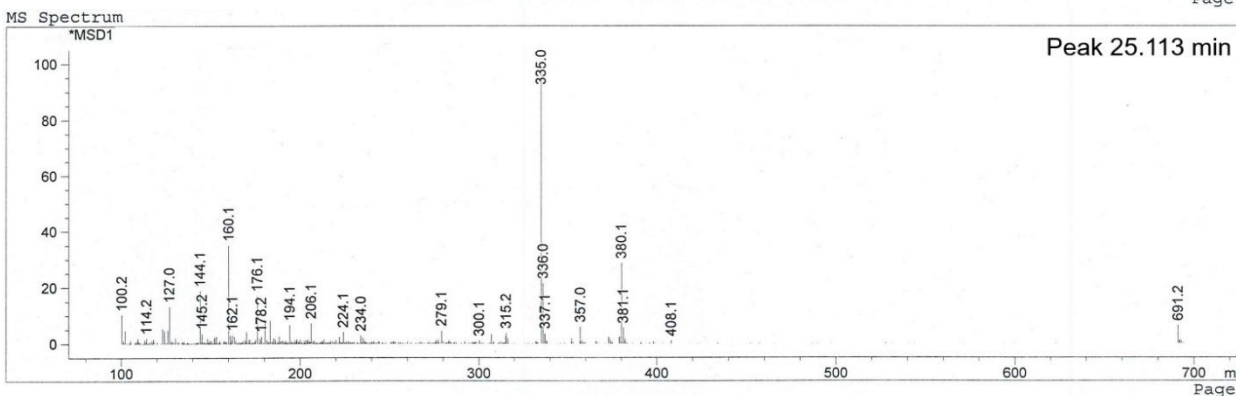

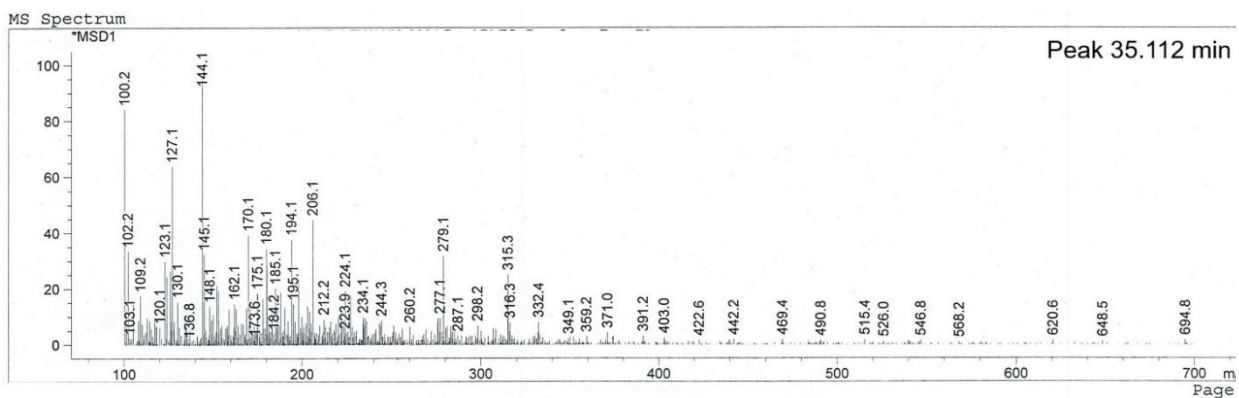

H

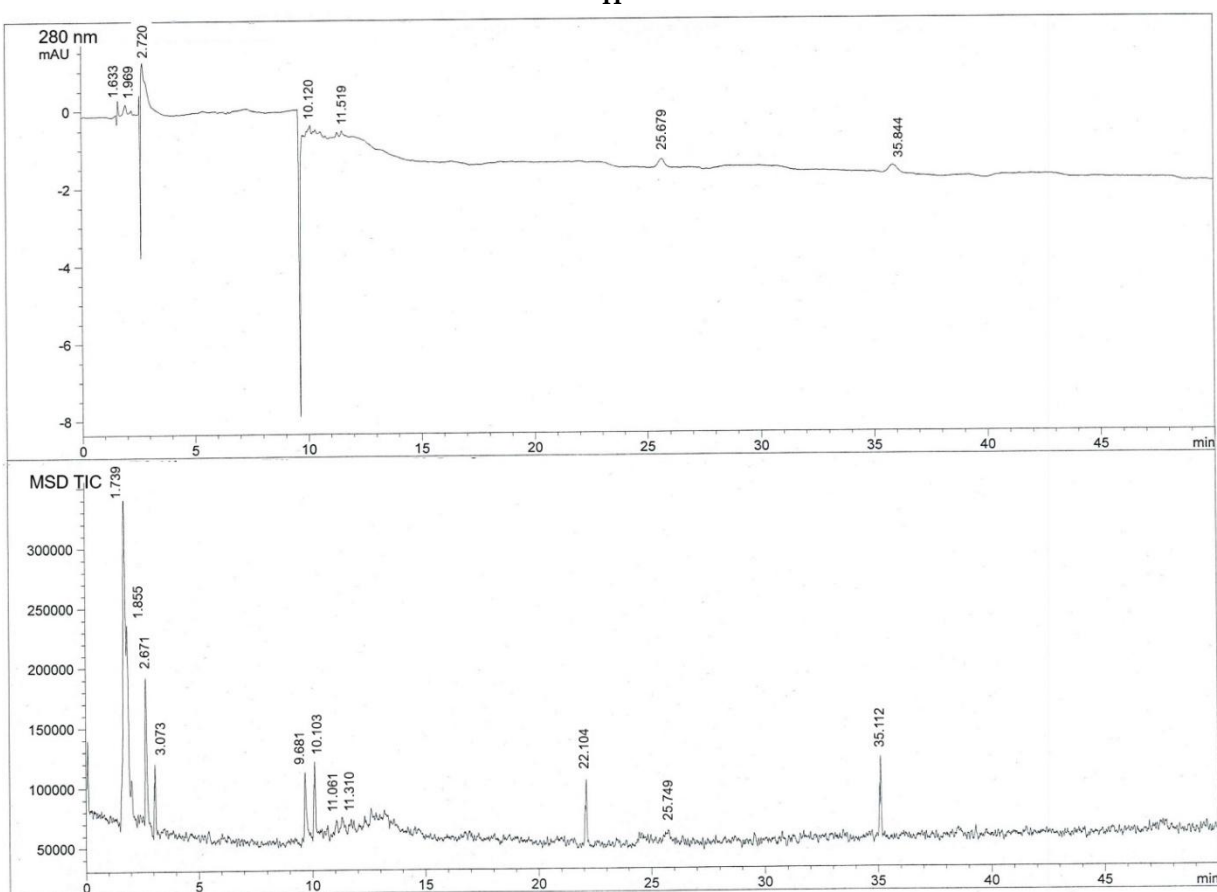

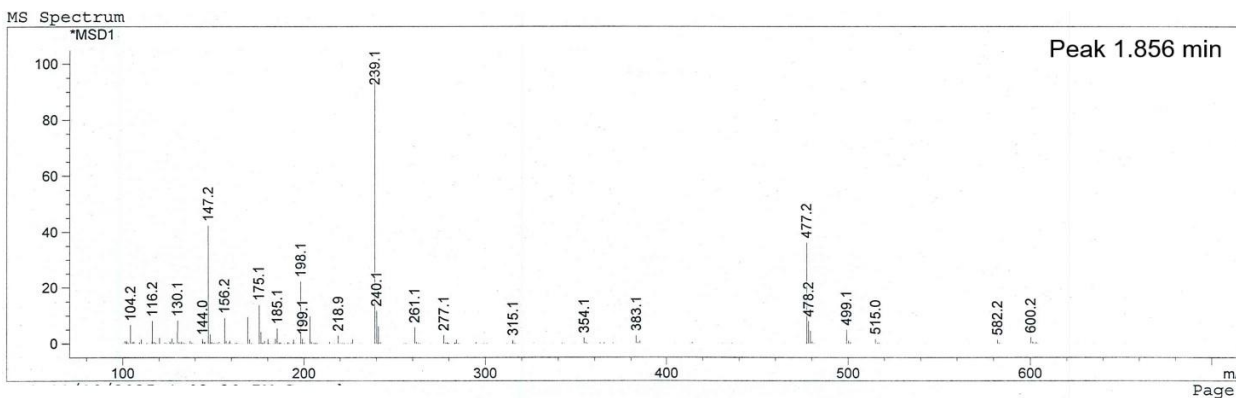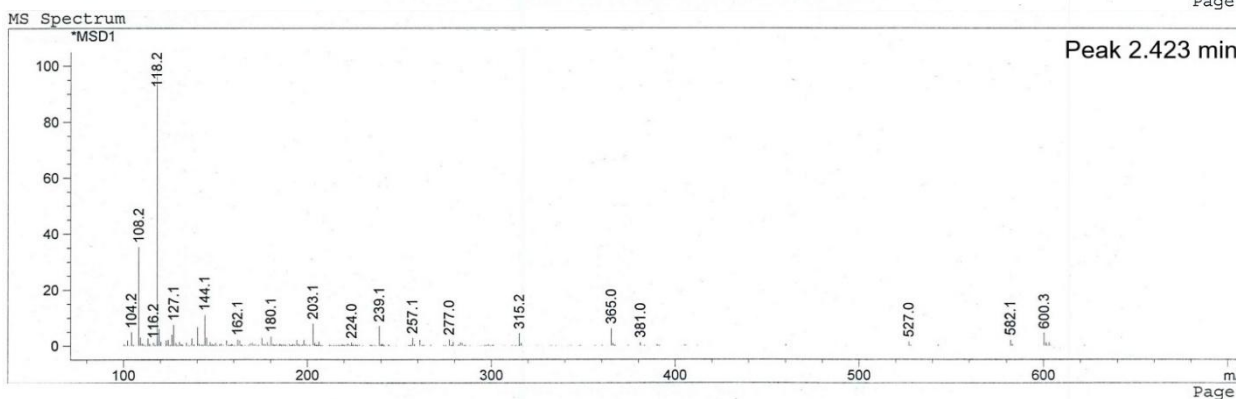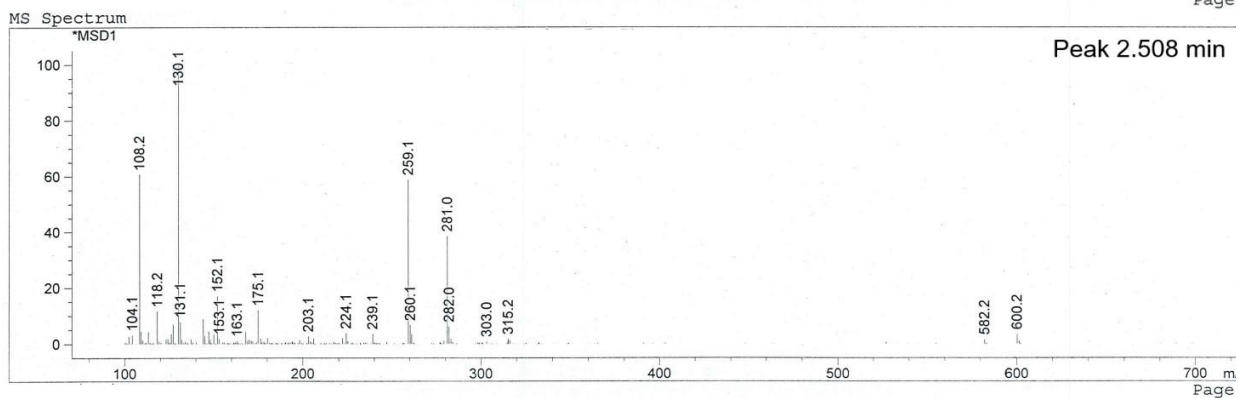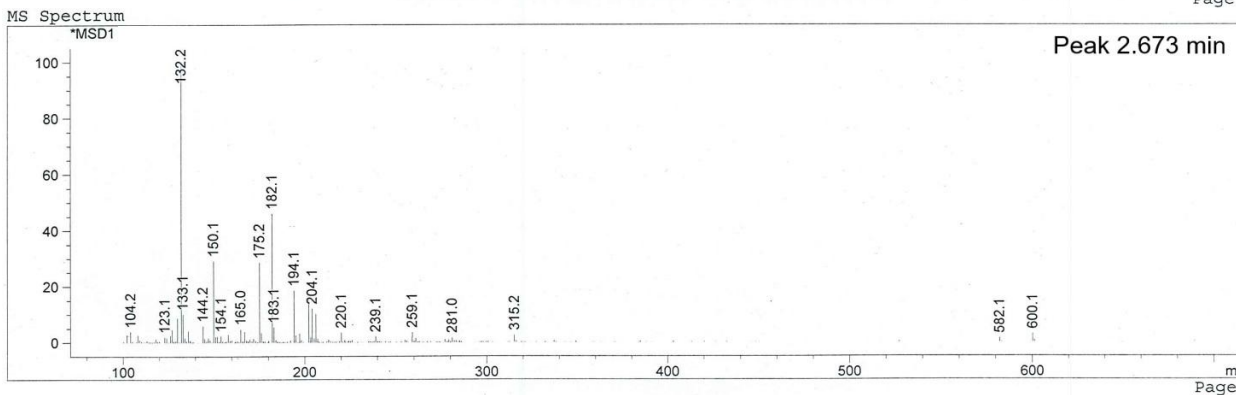

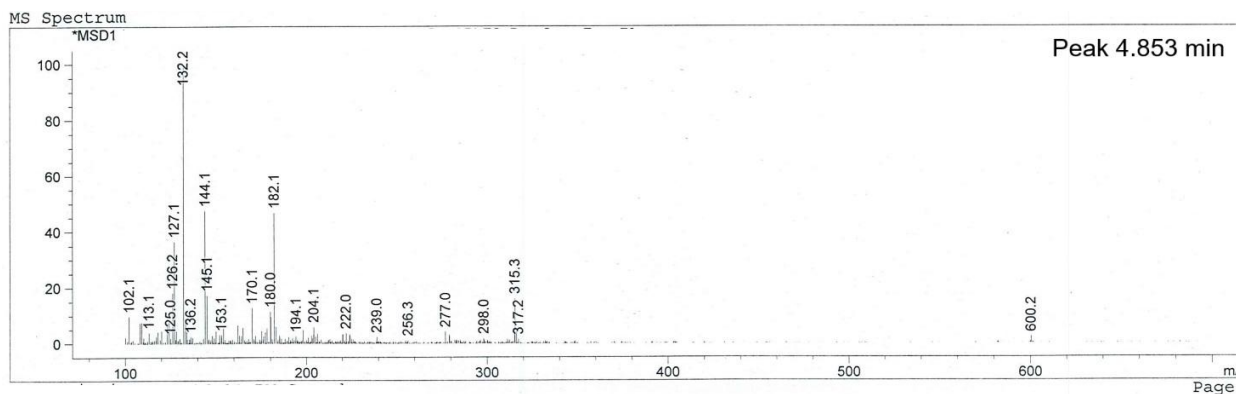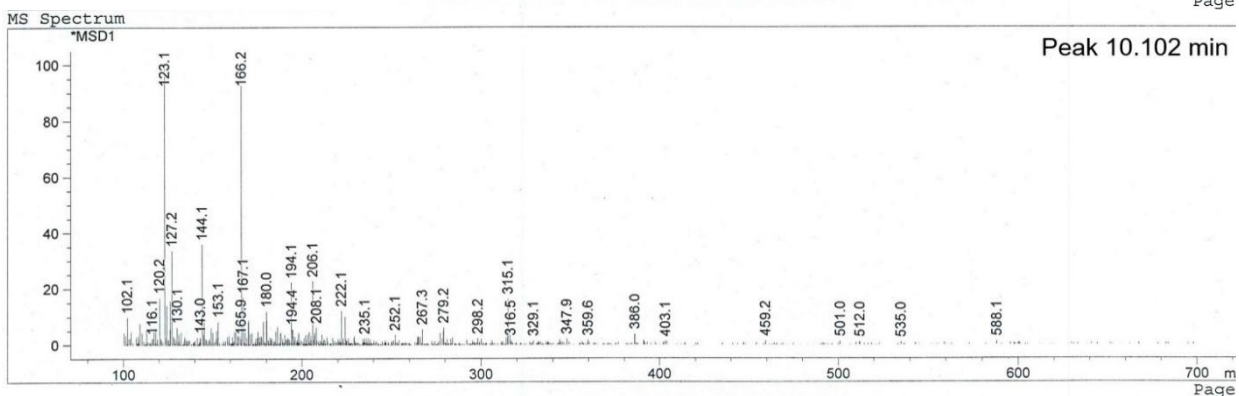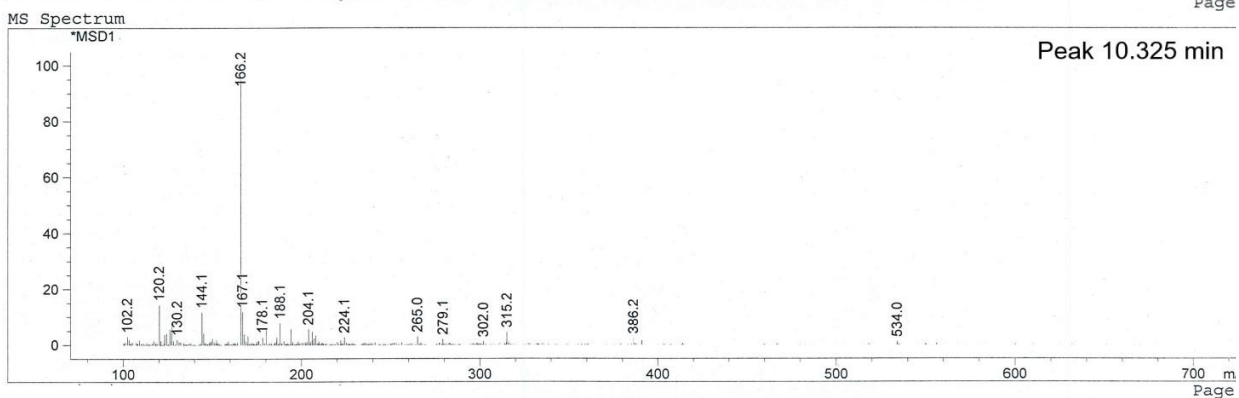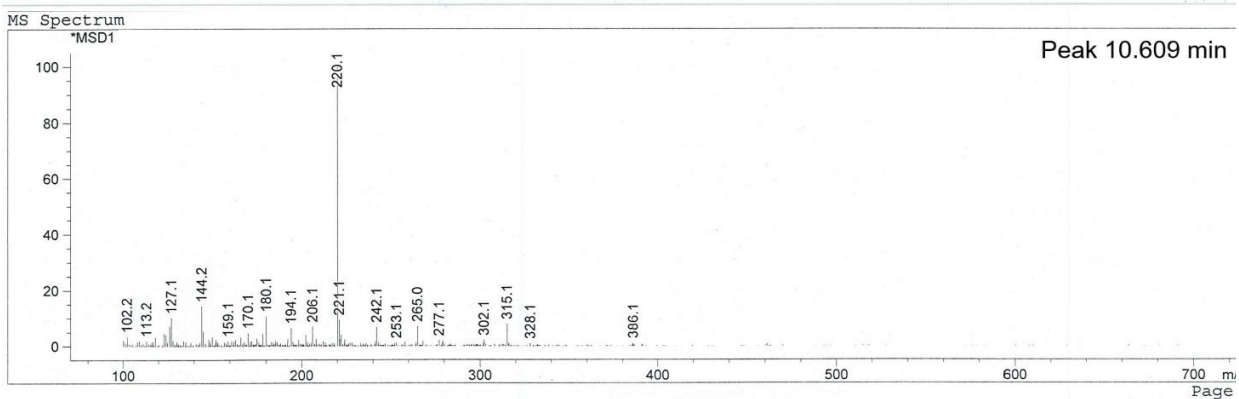

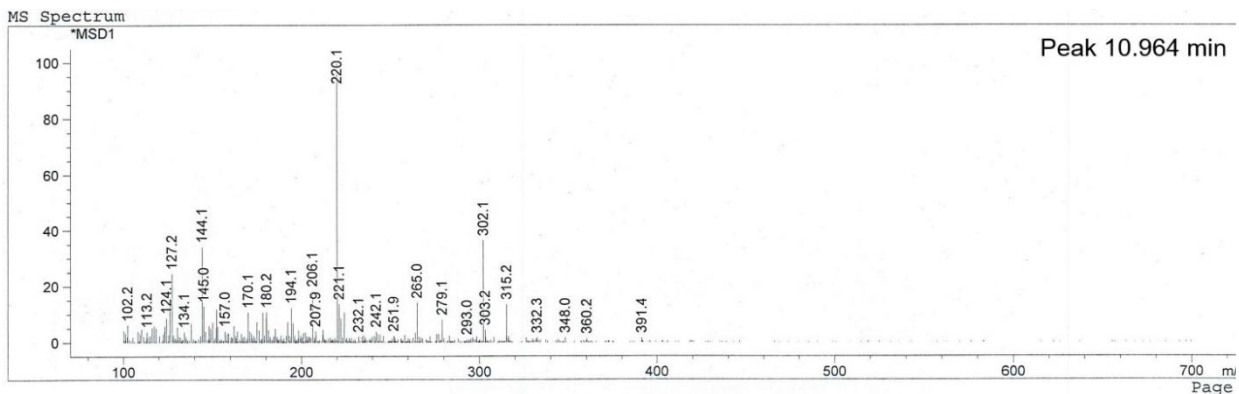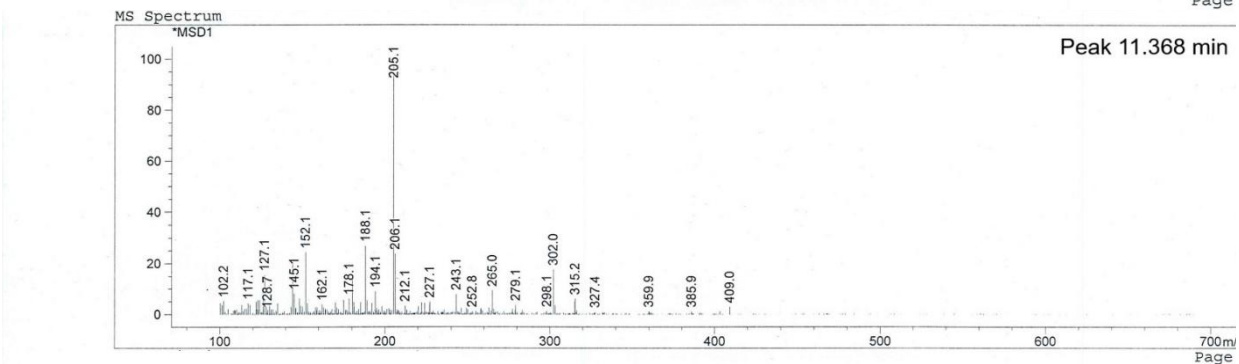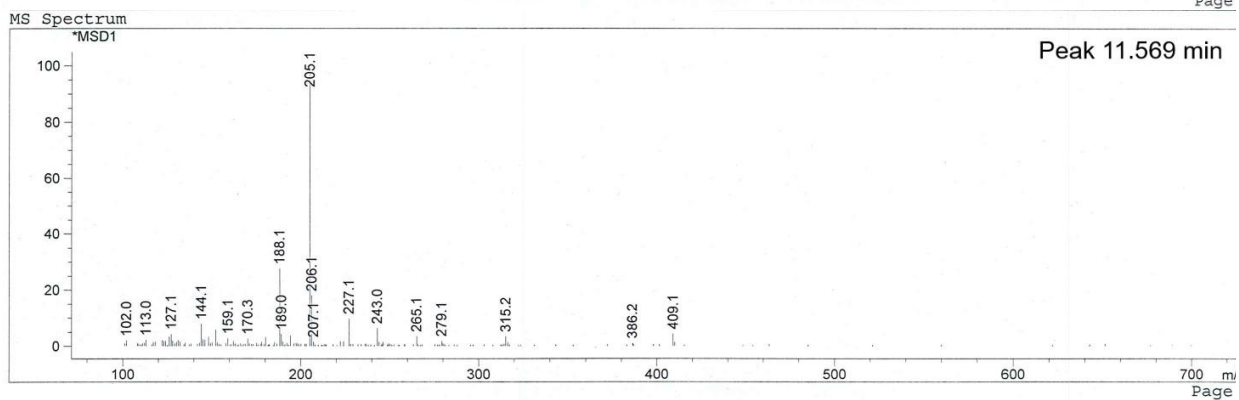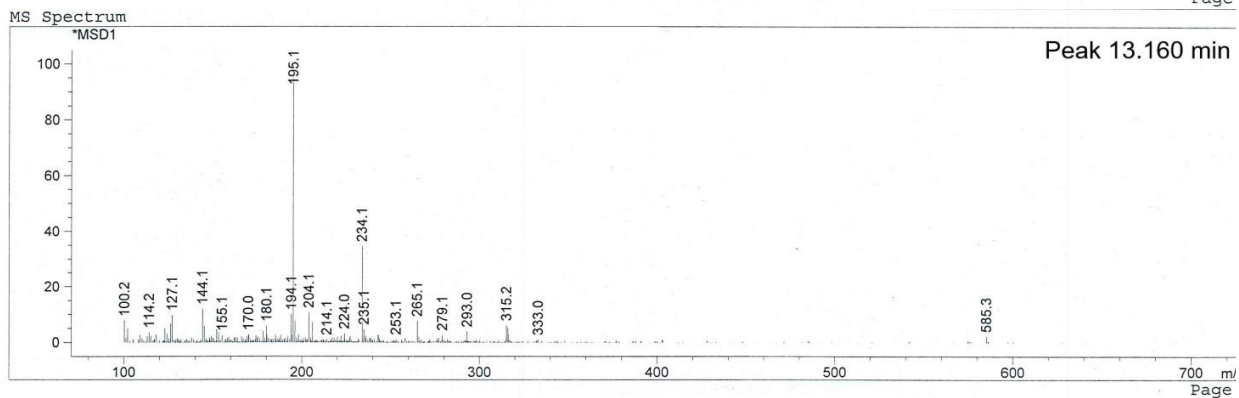

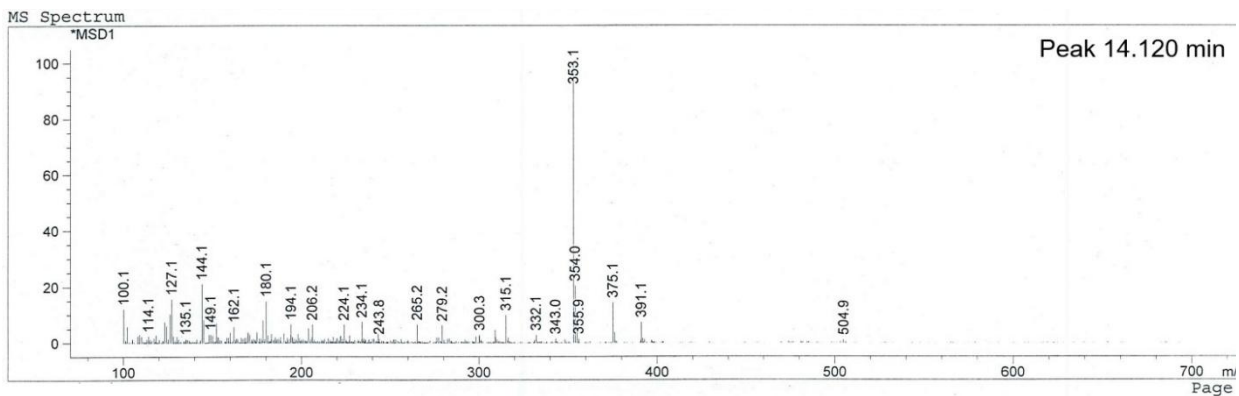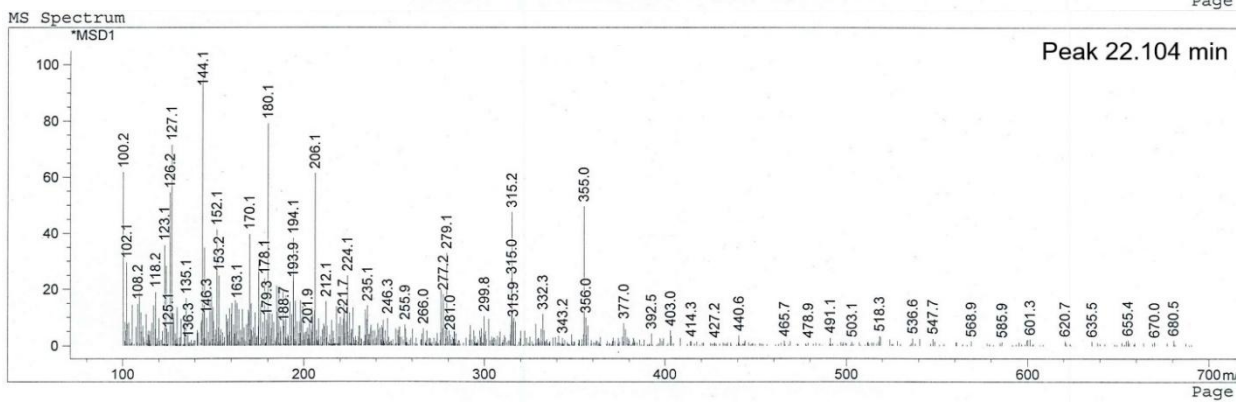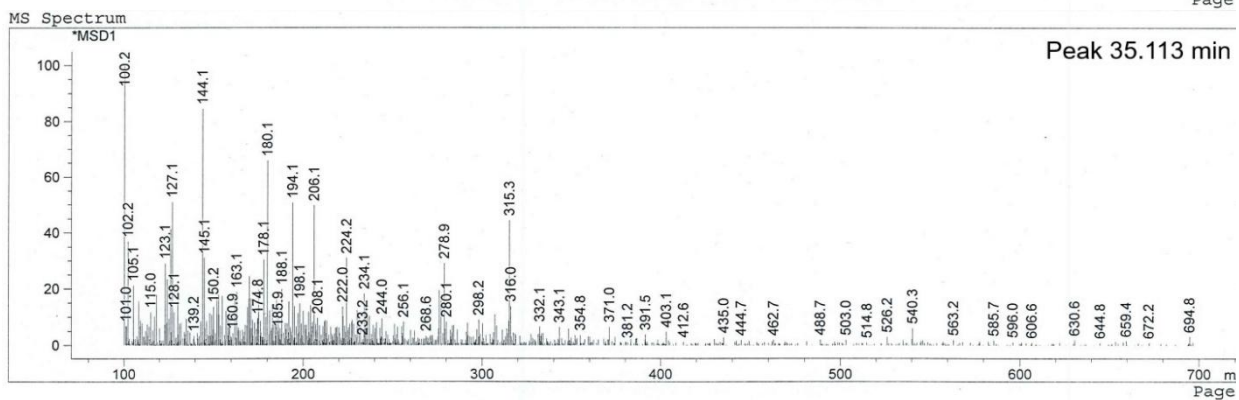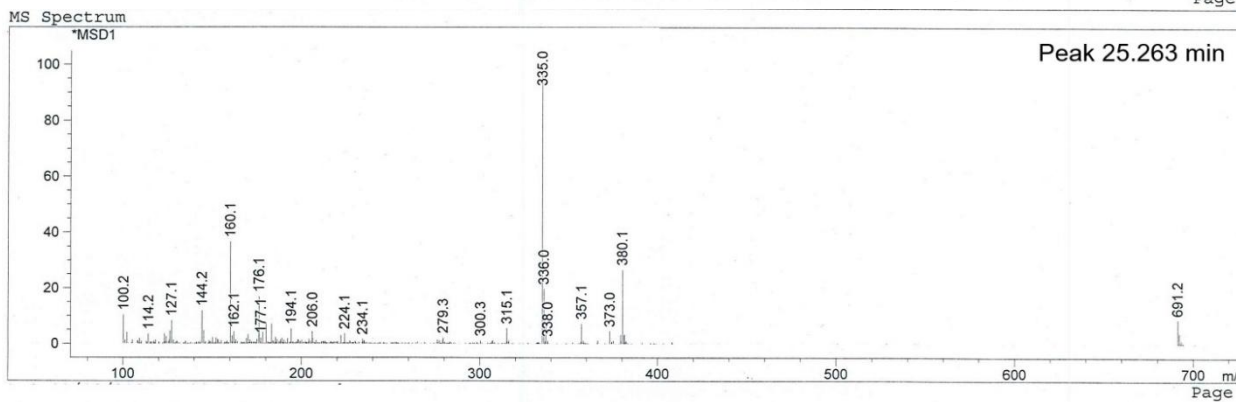

Supplement: Supplementary file 1 [file antioxidants-14-01483-s001.zip › antioxidants-3851199-supplementary.pdf]
